# Effects of heat-assisted sample desiccation on microbiome surveys

**DOI:** 10.1186/s40793-026-00889-5

**Published:** 2026-04-05

**Authors:** Claire E. Mullin, Stilianos Louca

**Affiliations:** 1 Department of Biology, University of Oregon, Eugene, USA; 2 Institute of Ecology and Evolution, University of Oregon, Eugene, USA

**Keywords:** metabarcoding, Metagenome-assembled genomes, Microbiome, Sample desiccation

## Abstract

Sample preservation remains a challenge in microbiome surveys, particularly in remote areas. Drying samples eliminates the need for cold chains and preservatives, but sophisticated desiccation tools such as lyophilization are impractical in the field. Further, the effects of sample drying on modern analyses, such as gene-centric metagenomics and metagenome-assembled genome (MAG) recovery, remain poorly understood. Here we explore heat-assisted sample desiccation followed by storage at room temperature as a cost-effective and practical solution in the field. We assess its effects relative to freezing on typical metagenomic and 16 S rRNA amplicon sequence analyses of bacterial and archaeal communities, using 60 samples from 6 different source materials (soils from 3 locations, feces from 3 animals). We consider multiple metrics related to the success of DNA extraction, sequencing, contig assembly, OTU clustering, gene annotation and MAG recovery, as well as impacts on inferred microbial community composition. We find that, while desiccation had a significant negative impact on multiple metrics related to DNA extraction success, its impacts on downstream metrics such as OTU richness, Shannon diversity, gene annotation and MAG recovery were more nuanced and often insignificant. Further, while the preservation method had a significant influence on the inferred microbial community composition, samples from different source materials (e.g., soils from different locations, or feces from different individuals) remained clearly distinguishable. We conclude that heat-assisted desiccation can be a viable sample preservation method for microbiome studies, when a high consistency with frozen samples is not a requirement.

## Introduction

The central role of microorganisms in the functioning of virtually all ecosystems, raging from soils to animal guts, has prompted intensive efforts to characterize microbial communities across biomes [60]. At the forefront of these efforts are modern molecular approaches such as metabarcoding (marker-gene amplicon sequencing) and metagenomics, which now permit the surveying of thousands of microbial taxa from minute quantities of material and even de novo construction of genomes without culturing. Nevertheless, preservation of samples remains a major logistical bottleneck during collection and transport, particularly in remote regions or in low-resource settings.

The prevailing sample preservation approach, maintenance of a cold chain, is often impractical. Alternative common strategies include (a) chemical fixation, for example using ethanol, and (b) desiccation [24, 42, 51, 64]. Chemical fixatives, on the one hand, present multiple drawbacks: most are classified as hazardous, they tend to substantially increase sample volume, can interfere with downstream molecular analyses, and generally afford only short-term stabilization because DNA degradation, though slowed, continues. Desiccation, on the other hand, allows sample preservation in a compact form and at room temperatures over prolonged periods, because in the absence of water most biochemical reactions essentially come to a halt. Sample desiccation also greatly facilitates shipping between countries as well as long-term archival of samples without the need for expensive and failure-prone freezers (see e.g., [39]). Lyophilization, for example, is a well-established desiccation method for preserving viable cells over multiple years [8, 26] and has also been suggested for preserving microbiome samples for sequencing [6, 39]. Unfortunately, lyophilization requires specialized equipment that is typically not available at field stations and even at some larger institutions, and is impractical to implement on most expeditions. Using desiccants such as silica at room temperatures is also problematic, due to their slow action increasing the risk of DNA degradation and sample spoilage [43, 44].

Heat-assisted desiccation may be a promising sample preservation approach that would resolve many of the aforementioned shortcomings. Indeed, it can be achieved using simple food dehydrators or block heaters, which are widely available even in less developed regions, it does not introduce additional chemicals to the samples, and is relatively safe and cheap. As a case in point, heat-assisted desiccation has recently been found to be an effective sample preservation method for hormone detection in wildlife fecal samples [32, 46]. Nevertheless, the effects of heat-assisted desiccation on microbial sequencing workflows, including 16 S rRNA metabarcoding and metagenomic sequencing, relative to common preservation methods such as freezing, have never been systematically evaluated. These effects may be substantial. Indeed, while heating accelerates sample desiccation, excessive heating can compromise the integrity of extracted DNA [3, 37, 45] or impact the attachment of other substances to DNA [20]. Further, heating and more generally desiccation could differentially affect different cell types [13, 57], thus altering the representation of taxa in the retrieved amplicon or metagenomic sequences.

Here, we examine heat-assisted desiccation as a technique for preserving microbiome samples collected in the field. Specifically, we use a custom-built block heater integrated into a compact carrying case and featuring sufficiently low power consumption for a typical 12V car power supply. We use this desiccator to compare the effects of sample desiccation followed by long-term storage at room temperature relative to freezing, on soil samples from 3 different locations and fecal samples from 3 domestic cats, at various stages of typical microbiome workflows. We note that our goal is not to determine the separate effect of every relevant factor, such as desiccation temperature, storage duration, DNA extraction protocol, bioinformatics parameters and so on. Instead, we focus on a few *typical* scenarios encountered in practice, to assess potential benefits and drawbacks of heat-assisted desiccation and estimate its effects on microbiome inferences compared to freezing and relative to other sources of variation such as sample heterogeneity. Further, we focus on bacteria and archaea (henceforth prokaryotes for brevity), which represent a common target in microbiome surveys. Workflows considered include 16 S rRNA metabarcoding for prokaryotes [60], gene-centric metagenomics for functional profiling [61] and recovery of metagenome-assembled genomes or MAGs [48]. We consider 20 quantifiable metrics of success, such as DNA extraction yield, absorbance ratios (representing DNA purity), peak fragment size (representing DNA quality), raw read counts, assembled contig sizes, numbers of recovered amplicon sequence variants (ASVs) and operational taxonomic units (OTUs) from amplicons (representing captured taxonomic diversity at different resolutions), and numbers of annotated genes, as well as the number and qualities of MAGs recovered from metagenomes. Further, we examine to what extent sample desiccation impacts inferred microbiome composition relative to freezing, stemming from potentially different biases in each technique. In addition, we examine to what extent samples from different environments or individual hosts remain clearly distinguishable following desiccation, which is an important criterion for comparative microbiome studies.

## Materials and methods

### Sample collection, desiccation and DNA extraction

For the purposes of this study and as a proof of principle, we constructed a portable and rugged heat-based sample desiccator, using easily available laboratory materials (photo in Fig. S1). The desiccator consists of four heated aluminum blocks kept at 60$^{\circ }$C by PTC elements controlled by low-cost thermostats, enclosed in a convenient carrying case and powered by a standard 12V car power supply (details in Supplement S.1.1). The desiccation temperature of 60$^{\circ }$C was chosen as a reasonable compromise between desiccation speed, which increases at higher temperatures, and DNA integrity, which starts decreasing substantially at temperatures around $\sim$75$^{\circ }$C [7]. To test the effects of this desiccation approach followed by sample preservation at room temperature, on common microbiome workflows and relative to conventional freezing, we collected soil materials from 3 different locations (an urban park, forest and grassland), as well as feces from 3 different domestic cats from distinct households (overview in Table S1 and Fig. S2). From each material we took 5 samples to be desiccated and subsequently stored at room temperature ($\sim$25$^{\circ }$C), as well as 5 samples to be frozen at -80$^{\circ }$C, until further processing. Freezing and drying were done on the same day, and thus this time point marks the beginning of divergent treatment between the two sets of samples. Replication was necessary in order to account for inevitable random effects in sample collection, DNA extraction and sequencing. Thus, in total 60 samples were analyzed, representing 6 different source materials. All samples were kept for 9 months until DNA extraction, followed by 16 S rRNA gene amplicon sequencing as well as (shotgun) metagenomic sequencing, described in more detail below. This duration was chosen as a representative of typical sample storage durations encountered in microbial ecology studies.

DNA was extracted from all samples using the same commercial kit (DNeasy^™^ PowerSoil Pro) and using the manufacturer’s standard protocol with minor adjustments (details in Supplement S.1.1). To examine the impact of heat-assisted sample desiccation relative to freezing on DNA extraction outcomes, we considered various metrics of “success”, including DNA yield (ng) measured using a Qubit^™^ 4 fluorometer, peak DNA fragment size (bp) as a measure of DNA integrity, and absorbance ratios at 260 nm/280 nm and 260 nm/230 nm as measures of purity.

### Statistical comparison of success metrics between treatments

**Fig. 1:**
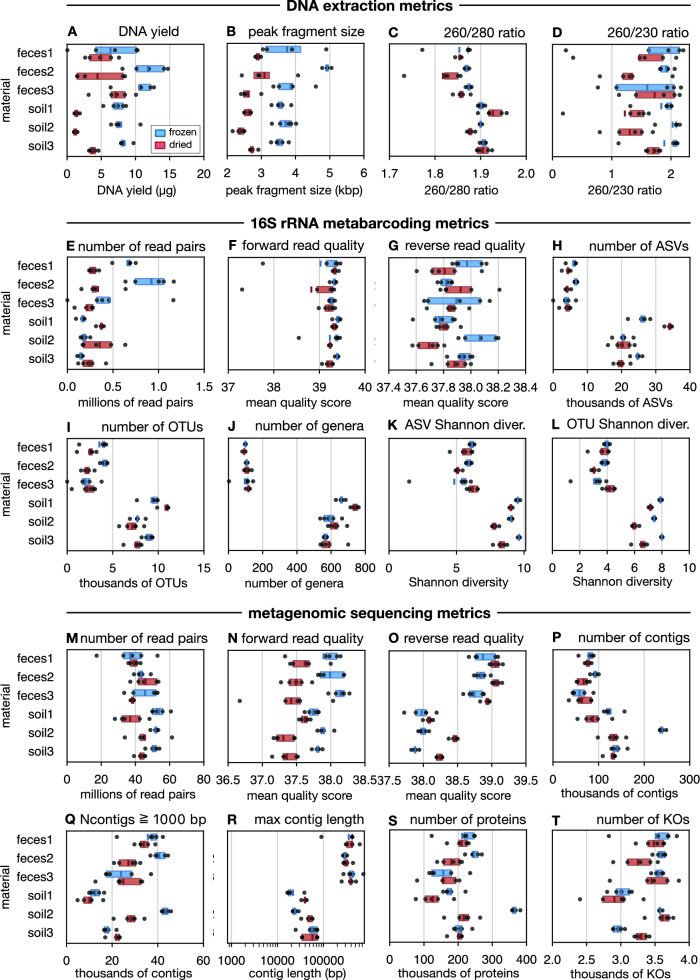
Success metrics by material and treatment. **A** Box-plots of DNA extraction yields (ng) obtained for each sample (one point per sample), visually grouped by material (feces 1–3, soils 1–3) and treatment (blue:frozen, red:dried). Boxes show interquartile ranges, small vertical bars show means. **B**–**P** Similar to A, but showing other metrics relevant to DNA extraction (top row), 16 S rRNA amplicon sequencing (second and third row, E-L) and metagenomic sequencing, assembly and gene annotation (bottom two rows, M-T). For statistical tests quantifying the differences between dried and frozen samples see Table S2

To quantitatively examine the effects of desiccation on the various considered success metrics, including the DNA extraction metrics mentioned above and success metrics related to 16 S rRNA metabarcoding and metagenomic sequencing described below, we statistically compared their average values between treatments (Fig. 1, Table S2). To assess statistical significance, we used a permutation model [22, 49] that assumes that for any given material both treatments are statistically equivalent, and which accounts for differences between source materials, described in detail in Supplement S.1.2. Briefly, for any given metric (e.g., DNA yield) we computed its average value for each material and treatment, then determined the ratio of these averages between treatments for each material (e.g., average DNA yield in dried soil 1 samples divided by the average DNA yield in frozen soil 1 samples), and then averaged those ratios across all materials. By forming the ratios separately for each material prior to averaging, we were able to control for general differences between materials. Note that an average ratio above or below 1 indicates an overall positive or negative effect of desiccation, respectively. We then computed the statistical significance of these average ratios with respect to the permutation null model using multiple datasets generated from the null model. Concretely, the null model permutes samples within each material across treatments, thus breaking any association that may exist between the metric and treatments.

### 16 S rRNA metabarcoding

Overview: To examine how sample desiccation affects typical short-read 16 S rRNA metabarcoding analyses, we performed amplification and Illumina sequencing of the V4-V5 region of the 16 S rRNA gene using the extracted DNA, described in detail below. As metrics of success, we considered the number of raw read pairs, the average Phred quality score of forward and reverse reads, the number of denoised prokaryotic amplicon sequence variants (ASVs), the number of recovered prokaryotic operational taxonomic units (OTUs, clustered at 99% identity), the number of identified prokaryotic genera, and the corresponding Shannon diversities at ASV and OTU level [50]. Further, we statistically assessed how the estimated proportions of ASVs, OTUs or genera differed between treatments.

16 S rRNA gene amplification and amplicon sequencing was performed for each sample by the Integrated Microbiome Resource (IMR) in Dalhousie, Canada. Specifically, the V4-V5 region of the gene was PCR-amplified using the Phusion Plus polymerase and “universal” bacterial + archaeal primers (515FB = GTGYCAGCMGCCGCGGTAA, 926R = CCGYCAATTYMTTTRAGTTT [47, 63]), and sequenced on a NextSeq 2000 (2$\times$300 bp paired ends). On average 361284 read pairs were obtained for each sample. Sequences were analyzed following Louca & Mullin [40], as follows. Reads were quality-filtered and amplicon sequence variants (ASVs) were inferred and chimera-filtered using the R package dada2 v1.28.0 [12]. Specifically, reads were first quality-filtered using the dada2 function filterAndTrim, with options “truncLen = (250,200), maxEE=(1,1), truncQ=(0,0), trimLeft=(6,6), minLen = (100,100), maxLen = (100000,100000)”, retaining on average 331398 read pairs per sample. Error model calibration for ASV inference was then performed jointly for all samples but separately for forward and reverse reads. Calibration was performed using the dada2 function learnErrors with options “nbases=1e8, randomize=TRUE, MAX_CONSIST=10, errorEstimationFunction=loessErrfun”. Reads were dereplicated using the dada2 function derepFastq. ASVs were inferred from dereplicated sequences separately for forward and reverse reads, using the dada2 function dada (options “pool=TRUE, selfConsist=FALSE”) and the previously calibrated error models. ASVs from forward and reverse reads were merged using the dada2 function mergePairs with options “minOverlap=15, maxMismatch=0, trimOverhang=TRUE”. Merged ASVs were chimera-filtered using the dada2 function removeBimeraDenovo (option method = ‘consensus’). ASVs were taxonomically classified based on a comparison to the SILVA database v138.2 [21], using a consensus approach [59]. Non-prokaryotic ASVs, including mitochondria and chloroplasts, were omitted from all subsequent analyses.

Many microbiome surveys are still conducted at the level of OTUs, and in fact ASVs (typically representing individual strains) and OTUs (operationally used as species-like units) can serve different purposes and each have advantages and disadvantages [19, 60]. We thus also clustered the retained ASVs de-novo into operational taxonomic units (OTUs) at 99% similarity, a commonly used species delineation threshold [18, 30], using vsearch –cluster_fast with options “–iddef 2 –strand plus”. This yielded an OTU table with 106,620 OTUs representing 9,325,236 reads across 60 samples. Sample metadata with 16 S rRNA success metrics are available as Data File 1.

**Fig. 2:**
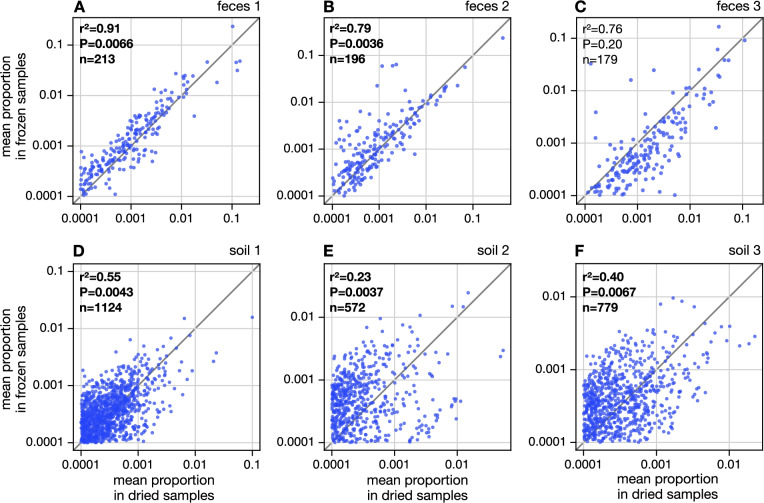
OTU composition vs. treatment. **A** Mean OTU proportions in dried fecal 1 samples (horizontal axis) compared to mean OTU proportions in frozen fecal 1 samples (vertical axis, one point per OTU), considering only OTUs with proportion $\ge$0.01% in at least one sample. The diagonal is shown for reference. Inscriptions show the Pearson correlation between the log-transformed mean OTU proportions in frozen and dried samples ($r^2$), the number of OTUs considered (*n*), and the statistical significance of $r^2$ compared to a permutation null model under which OTU proportions are statistically indistinguishable in the two treatments (**P**). Note that this null model differs from the conventional null model of zero correlation. A significantly low $r^2$ $(i.e., \hbox {P}<0.05)$ suggests that dried samples tend to yield different OTU proportions compared to frozen samples. **B**–**F** Similar to A, but for each of the other source materials. Statistically significant $r^2$ values are boldened. For similar plots that also include rare OTUs (proportions <0.01%) see Fig. S3. For similar plots using ASV or genus proportions see Fig. S4 and S6, respectively

To further examine how desiccation impacts estimated community composition relative to freezing, we compared the proportions of OTUs (and similarly for ASVs and genera) between treatments while controlling for the material (mathematical details in in Supplement S.1.3). Briefly, for each material and each OTU, we calculated the OTU’s mean proportion across all 5 dried samples and separately across all 5 frozen samples, and then scatterplotted these mean proportions on two axes (Figs. 2, S3, S4, S5 and S6). We measured the agreement between treatments in terms of the Pearson correlation of the log-transformed mean proportions across OTUs ($r^2$). Thus, a large $r^2$ would indicate a strong consistency between the two treatments — in the ideal case, and in the absence of any stochastic effects, OTUs should have the same mean proportions in dried as in frozen samples, that is, all points should be on the diagonal, and the Pearson correlation should be equal to 1. We stress that in reality, in addition to potential treatment effects, various stochastic effects could also lead to differences in inferred OTU proportions between samples, and thus an observed correlation coefficient below 1. Examples of such effects include random effects in PCR and sequencing, as well as microbial content differences between replicate samples collected across a heterogeneous material. Thus, the observed inconsistencies in mean OTU proportions between treatments need not necessarily be due to the treatment. To resolve this uncertainty, we tested the plausibility of the observed $r^2$ under a permutation null model that assumes no effects of treatment but controls for material and accounts for general stochastic effects (details in in Supplement S.1.3). Specifically, the null model randomly shuffles samples across treatments while maintaining their material identity, thus breaking any associations that may exist between OTU proportions and treatment (results embedded in Figs. 2, S3, S4, S5 and S6). The statistical significance of the observed $r^2$ was set to the probability that the null model would generate a lower $r^2$ (i.e., lead to a lower consistency between treatments) by chance.

**Fig. 3:**
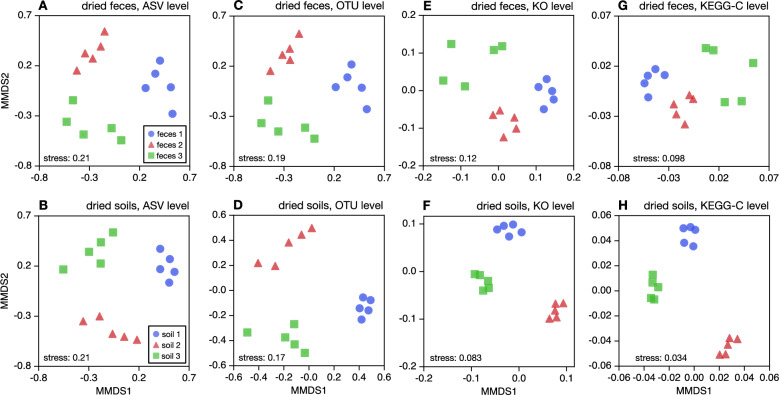
Community differences between dried samples. **A** Metric multidimensional scaling (MMDS) plot of weighted Bray-Curtis dissimilarities between dried fecal samples, based on ASV proportions. Points correspond to samples, and are shaped and colored according to the source material (feces 1–3). The Kruskal stress is written in the plot.** B** Similar to A, but for dried soil samples. **C**,**D** Similar to A,B but showing dissimilarities in terms of OTU proportions. **E**,**F** Similar to A,B, but showing dissimilarities in terms of gene proportions. **G**,**H** Similar to A,B, but showing dissimilarities in terms of KEGG C gene group proportions. These figures show that drying clearly preserves the signal of source material. For MMDS plots overlaying both frozen and dried samples, see Figures S8 and S11

To further examine the contribution of treatment and source material to differences in estimated taxonomic composition, we considered the pairwise dissimilarities between samples in terms of inferred ASV, OTU or genus proportions. Pairwise dissimilarities were computed using the well-established abundance-weighted Bray-Curtis metric, which weighs contributions of taxa by their relative abundances [14, 35]. Two-dimensional metric multidimensional scaling (MMDS) embeddings based on these pairwise dissimilarities, for various sample subsets discussed in the main text, were generated by minimizing the Kruskal stress function [9] (Figs. 3, S7 and S8). To assess the extent to which drying or freezing affected inferred taxonomic compositions, PERMANOVA tests were conducted on these dissimilarities, using the pseudo F test statistic and estimating statistical significances through 10000 permutations [4]. A significantly high pseudo F statistic indicates that pairwise dissimilarities between treatments tend to be detectably larger than within each treatment (Table S3). Similar PERMANOVA analyses were also conducted to assess the effects of the source material on pairwise dissimilarities (Tables S4, S5).

### Metagenomics

Overview: To examine the effects of sample treatment on typical metagenomic workflows, we also performed short-read paired-end Illumina metagenomic sequencing of all samples, described in detail below. Our workflow included assembly of reads from individual samples into longer contiguous sequences (contigs), as well as coassembly of reads after pooling samples from the same treatment and source. Similarly to the metabarcoding analysis above, we considered various metrics of success, including the number of obtained raw read pairs, the mean quality score of forward and reverse reads, the number of assembled contigs, the number of assembled contigs at least 1000 bp long, the maximum assembled contig length, the number of protein-coding sequences predicted in the assembled contigs and the number of annotated genes (KEGG Orthologs, or KOs). Further, we statistically assessed how the proportions of genes (KOs) or gene groups (KEGG level C), estimated from the coassembled contigs, differed between treatments.

Metagenomic sequencing was performed by the University of Minnesota Genomics Center, using the 1/4 Reaction Nextera XT Library Prep kit and a NovaSeq X Plus sequencer (3$\times$150 bp read pairs). On average 44248833 metagenomic read pairs were obtained per sample. Sequences were analyzed following Louca & Mullin [40], as follows. Adapters were trimmed from reads using skewer v0.2.2 [28]. Reads were quality-filtered using vsearch v2.28.1 [52] with options “–fastq_ascii 33 –fastq_maxee 0.5 –fastq_truncee 0.5 –fastq_qmax 64 –fastq_maxee_rate 0.002 –fastq_stripleft 0 –fastq_trunclen_keep 10000 –fastq_minlen 100”, keeping on average 22,320,196 high-quality read pairs per sample. Quality-filtered paired reads from each sample were then assembled into longer contiguous sequences (contigs) using megahit v1.2.9 [36] with option “–min-contig-len 500”. On average 107,637 contigs were generated per sample. Protein-coding genes (PCGs) were predicted in the contigs using prodigal v2.6.3 with option “-p meta” and otherwise default options [27]. PCGs were then mapped to KEGG gene orthologs (KOs) in the KOfam HMM database r111 [5] using hmmsearch v3.3 [41]. Only hits with an E-value below $10^{-10}$ were considered. Various aspects of the assemblies, such as the maximum contig length, the number of contigs and the number of detected KOs, were considered as success metrics (Fig. 1M-T).

Quality-filtered paired reads from multiple samples were also coassembled separately for each combination of treatment and source type (i.e., dried feces, frozen feces, dried soils, frozen soils). Note that coassembly of related samples generally yields longer contigs and enables the use of differential coverages to improve MAG recovery [2, 15]. We deliberately did not pool samples from different treatments for coassembly, for two reasons: First, our objective was to compare outcomes (such as MAG recovery) independently for each treatment, i.e., ensuring that different treatments do not influence each other’s performance in any way. Second, coassembly across treatments would make our analyses less translatable to realistic scenarios, as most studies deploy only one sample preservation method at a time. Gene-centric functional profiles for each sample were computed from these coassembled contigs similar to [40, 58, 59], as follows. Contig coverages were computed for each sample by mapping the non-assembled reads of that sample to the contigs, then calculating the number of reads mapped per contig with a MAPQ score $\ge$30 and dividing that number by the length of the contig. Contig coverages were then normalized such that they sum to 1 in each sample, thus yielding contig “proportions” for each sample. PCGs were then detected in the contigs using prodigal [27] and mapped to KOs using hmmsearch [41], as described earlier for assemblies. Proportions of PCGs were computed in each sample by first associating with each PCG the proportion of its host contig, and then normalizing those values in each sample to sum 1. The proportion of a given gene in a given sample was estimated by summing the proportions of all PCGs mapped to the gene. KOs were also grouped into standard high-level functional groups at KEGG level C; the proportion of each group in each sample was computed as the sum of proportions of all associated genes (Fig. S9). The following KEGG groups were omitted, as they are not actually defined based on function: “brite hierarchies”, “enzymes with ec numbers”, “not included in pathway or brite”, “poorly characterized”, “general function prediction only”, “others”, “unclassified viral proteins”, “function unknown”. Sample metadata with metagenomic success metrics are available as Data File 2. All analyses of gene proportions were based on the coassemblies.

**Fig. 4:**
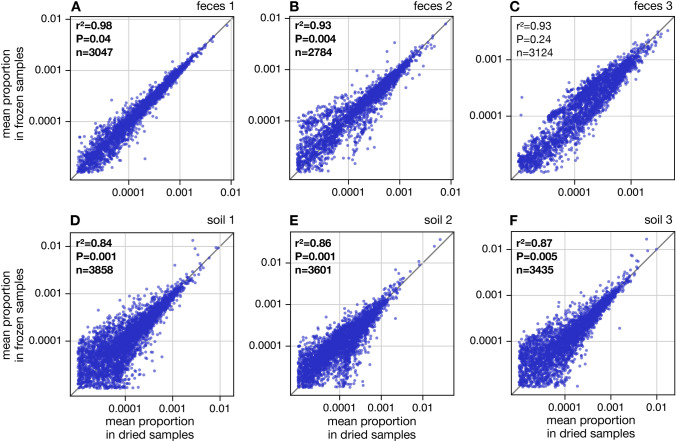
Gene composition vs. treatment. **A** Mean gene (KO) proportions in dried fecal 1 samples (horizontal axis) compared to mean gene proportions in frozen fecal 1 samples (vertical axis, one point per gene), considering only KOs with proportion $\ge$0.001% in at least one compared sample. Averaging of proportions was done among the 5 replicates in each treatment. The diagonal is shown for reference. Inscriptions show the Pearson correlations between the log-transformed mean gene proportions in frozen and dried samples ($r^2$), the number of genes considered (*n*), and the statistical significance of $r^2$ compared to a permutation null model under which gene proportions are statistically indistinguishable in the two treatments (P). A significantly low $r^2$ $(i.e., \hbox {P}<0.05)$suggests dried samples tend to yield different gene proportions compared to frozen samples. **B**–**F** Similar to A, but for the remaining samples. Statistically significant $r^2$ values are boldened. For similar plots considering gene group proportions at KEGG level C see Fig. S10

Pairwise dissimilarities between samples in terms of gene (KO) or gene group (KEGG C) proportions were computed using the abundance-weighted Bray-Curtis metric [35]. MMDS and PERMANOVA analyses of those dissimilarities were conducted as described above for OTUs (Tables S6, S7, S8). Further, gene and gene group proportions were visually and statistically compared between treatments using an approach similar to OTUs, as described earlier (Figs. 4 and S10). To avoid excessive noise associated with sampling stochasticity, only genes or gene groups with a mean proportion of at least 0.001% in at least one compared sample were considered for this analysis.

**Fig. 5:**
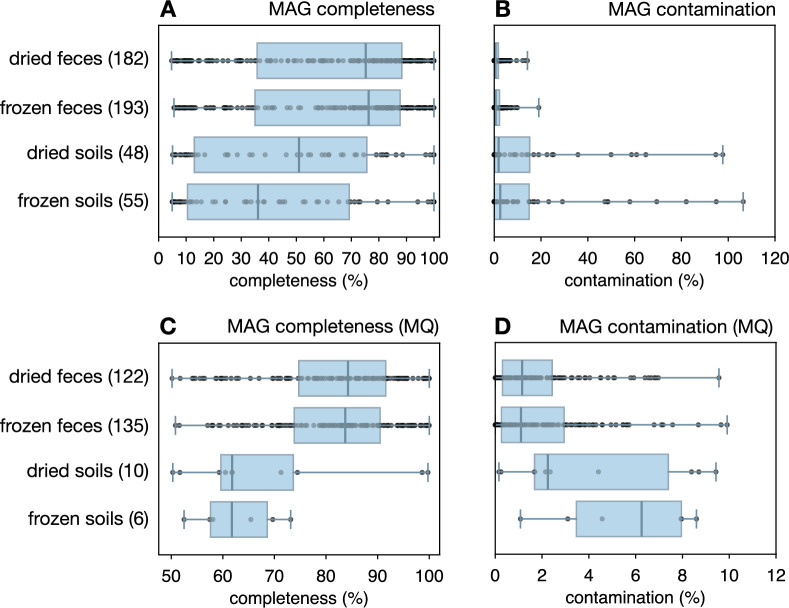
MAG qualities. **A** Box-whisker plots of MAG completeness levels, separately for each combination of source type (feces or soil) and treatment (dried or frozen). The number of MAGs is written in the box labels. Boxes show interquartile ranges, whiskers show full data ranges, points correspond to individual MAGs, and vertical bars show medians. **B** Similar to A, but showing MAG contamination levels. **C**,**D** Similar to A,B, but focusing on MAGs of at least medium quality (completeness $\ge$50% and contamination $\le$10%). For average completeness and contamination levels, see Table S9

Coassembled contigs were subsequently binned into metagenome-assembled genomes (MAGs), separately for each combination of treatment and source type. Note that binning MAGs concurrently from multiple samples — as opposed to binning from individual samples — is generally recommended and standard practice, as this allows the incorporation of differential coverage profiles in the binning process, which in turn tends to improve MAG quality [2, 15]. Partitioning our samples into combinations of treatment (dried vs. frozen) and source type (feces vs. soil) allowed us to assess the effects of treatment while controlling for type. For each sample, reads were mapped back to the contigs from the corresponding coassembly using bowtie2 v2.5.1 [34] with option “–no-unal”. SAM files created by bowtie2 were converted to BAM format using samtools v1.17. BAM files and contig fasta files were then used as input to MetaBAT2 v2.15-2 [29] for MAG recovery, with options “-m 3000 -s 200000”. This yielded a set of MAGs for each coassembly, that is, for each combination of treatment and source type. MAG completeness & contamination were estimated using checkM2 v1.0.1 [16]. Overviews of recovered MAGs are shown in Fig. 5 and listed in Table S9.

## Results and discussion

### Effects on DNA extraction

To examine the impact of heat-assisted sample desiccation relative to freezing on DNA extraction outcomes, we extracted DNA from all samples using the same commercial kit and assessed DNA yield (ng), peak DNA fragment size (bp) as a measure of DNA integrity, and absorbance ratios at 260 nm/280 nm and 260 nm/230 nm as measures of purity. A simple visualization of these metrics, separately for each source material (feces 1–3, soils 1–3) and separately for each treatment (dried vs. frozen), indicates that desiccation had a negative impact in most cases (Fig. 1). For example, the average DNA yield from dried soil samples was less than half of the average DNA yield from frozen soil samples, and this was separately true for each of the 3 soil materials (Fig. 1A). Desiccation also tended to substantially decrease the peak DNA fragment size compared to their frozen counterparts. This reduction in DNA yield and peak fragment size may be due to stress and shear experienced by DNA under heating [7]. Similarly, 260/230 absorbance ratios dropped substantially in dried soil samples compared to frozen ones. This indicates that heat-drying alters soil chemistry and organic matter in ways that promote co-extraction of 230 nm–absorbing compounds, such as humic and fulvic acids. Indeed, previous work has shown that heating soils tends to increase humic and fulvic acid concentrations [31, 65]. A weak positive effect was only observed in isolated cases, most notably in the 260/280 absorbance rations of soil 1 samples (Fig. 1C).

Next, we statistically examined the effects of desiccation on each of the above metrics by dividing its average values among dried samples by its average values among corresponding frozen samples, and then evaluating the statistical significance of the average ratio relative to a permutation null model (see Methods for details). Note that an average ratio above or below 1 indicates an overall positive or negative effect of desiccation, respectively. With the exception of the 260/280 ratio, for which no significant effect was detected, all other metrics exhibited a statistically significant reduction in dried samples $(P<0.05)$, with DNA yield exhibiting a particularly low ratio of 0.438, followed by peak fragment size with a ratio of 0.705 (detailed results in Table S2). This means that, on average, desiccation followed by storage at room temperature reduced DNA yield by more than 50% and peak fragment size by nearly 30%, compared to freezing. For short Illumina amplicon sequencing, such as metabarcoding of the 16 S rRNA V4-V5 region, this reduction in DNA yield and peak fragment size may only have a modest downstream impact, and it is more likely to impact data-hungry genome-resolved metagenomics workflows or long-read sequencing techniques such as Nanopore and PacBio.

### Effects on 16 S rRNA metabarcoding

#### Effects on metabarcoding success metrics

Next, we examined the effects of desiccation on a total of 8 success metrics related to 16 S rRNA metabarcoding, such as the number of read pairs, the average quality scores of reads, the number of recovered prokaryotic ASVs, OTUs or genera and Shannon diversities at ASV and OTU level (details in Methods). Based on a visual assessment, in most cases we did not observe any clear directional impact of desiccation, which sometimes improved and sometimes deteriorated the outcome (Figs. 1E–L). For example, while dried samples generally yielded a lower number of reads for fecal samples, the reverse was the case for soil samples (Fig. 1E). To quantitatively examine the effects of drying on the above metrics relative to freezing, we also performed statistical comparisons between treatments similarly to those described earlier (detailed results in Table S2). A statistically significant effect was only detected for the quality scores of reverse reads, which was slightly lower for dried samples (average ratio 0.998). While dried samples yielded on average 19% more reads, 0.2% lower forward read quality scores, 7% fewer ASVs, 13% fewer OTUs, 7% more genera, 5% lower ASV Shannon diversities and 7% lower OTU Shannon diversities, none of these effects were statistically significant.

#### Effects on inferred community composition

To examine how treatment impacts estimated community composition, separately for each source material, we calculated each OTU’s mean proportion across all 5 dried samples and separately across all 5 frozen samples, and then scatterplotted these mean proportions on two axes (Fig. 2 and Fig. S3). We then computed the Pearson correlation coefficient ($r^2$) between these average OTU proportions and assessed its statistical significance (*P* value) relative to a permutation null model in which treatment has no effect on the distribution of OTU proportions (details in Methods; $r^2$ and *P* values shown in Figs. 2 and Fig. S3). Thus, a lower correlation coefficient between the two treatments is indicative of a stronger treatment effect and will generally tend to be more statistically significant. We observed a modest to strong consistency between treatments depending on the material, with the strongest consistency seen for feces 1 ($r^2=0.91$) and feces 2 ($r^2=0.79$) and the lowest consistency seen for soil 2 ($r^2=0.23$) and soil 3 ($r^2=0.40$). For nearly all materials with the exception of feces 3, the correlation was significantly lower than expected under the null model ($P<0.05$). This was true both when considering only abundant OTUs, i.e. with proportion $\ge$0.01% in at least one compared sample (Fig. 2), and when including rare OTUs as well (Fig. S3). Thus, even for those materials where a high correlation was observed (e.g., feces 1), this correlation was still significantly worse than expected purely by chance. This suggests that the treatment has had a statistically detectable impact on inferred OTU compositions, even if at times this impact was small. Similar results were obtained at the resolution of ASVs (roughly representing strains, Figures S4 and S5) and genera (Fig. S6). These taxonomic inconsistencies may be caused, for example, by different impacts of drying and freezing on cell rupture prior to and during DNA extraction, in turn impacting the representation of taxa in the DNA sequences. Indeed, some cell types may resist degradation during desiccation more than others, or may be preserved at room temperature after desiccation better than others, or may be easier to lyse during DNA extraction after having been dried or frozen.

#### Effects on taxonomic $\beta$-diversity

Next, we wanted to test whether dried samples can be reliably distinguished in terms of their source material, based on the common Bray-Curtis dissimilarity ($\beta$ diversity) metric [35]. This question is particularly important for comparative microbiome studies, where the goal is often to detect microbiome differences between samples exposed to different experimental or environmental conditions. To this end, we visualized pairwise dissimilarities computed from ASV or OTU proportions, using metric multidimensional scaling (MMDS) plots [9], separately for dried fecal samples and dried soil samples (Fig. 3A–D). This revealed that samples from different feces are clearly discernible from each other, that is, they form distinct clusters in the MMDS plot, both at the level of ASVs and OTUs. This observation is quantitatively supported by our PERMANOVA tests [4], which statistically compare the separation within materials to the separation between materials (Table S5). Indeed, we found a strong and statistically significant separation between materials, both for dried feces and for dried soils (*P* < 0.001 in all cases), with material differences explaining between 64% and 77% of the variance in dissimilarities depending on the specific case. Thus, a hypothetical study using exclusively desiccation for sample preservation would have no difficulties detecting microbial composition differences between the three fecal materials or between the three soil materials.

Nevertheless, when we visualized pairwise dissimilarities using MMDS separately for each material (for example, only feces 1 samples), we also observed a clear clustering of samples by treatment (Fig. S7). This clustering was quantitatively confirmed using PERMANOVA tests at all considered taxonomic levels, i.e., ASVs, OTUs and genera (Table S3). Indeed, for any given source material, PERMANOVA revealed a statistically significant separation of samples by treatment (*P* < 0.05), and in many cases the treatment explained over 50% of the variance in pairwise dissimilarities. Thus, consistent with our earlier analysis of correlations between taxon proportions, we find that treatment has a detectable impact on prokaryotic taxonomic beta diversity.

Lastly, to compare the relative effects of treatment and material on taxonomic composition, we also visualized the pairwise dissimilarities using MMDS for all fecal samples (i.e., both dried and frozen feces 1–3) and all soil samples (Fig. S8), and conducted corresponding PERMANOVA analyses that either compare treatments or source materials (Table S4). Consistent with our earlier results, we detected a significant separation both by treatment as well as by material, at all considered taxonomic levels (ASV, OTU and genus). However, the fraction of variance in pairwise dissimilarities explained by the treatment was almost always much smaller than what could be explained by the source material (Table S4). For example, at OTU level treatment explained 4.2% and 22% of the variance for feces and soils, respectively, whereas source material explained 49% and 46% of the variance for feces and soils, respectively. Similarly, the standardized effect sizes were almost always much smaller when comparing treatments than when comparing materials, the only exception being soils at the genus level. Thus, the effects of the source material on ASV and OTU differences between samples appeared to be much stronger than the effects of the treatment, while at the genus level the difference was more ambiguous.

### Effects on metagenomics

#### Effects on metagenomic success metrics

Next, we performed Illumina metagenomic sequencing of all 60 samples and examined various related metrics of success, such as the number of raw read pairs, mean quality scores of reads, the number of assembled contigs, the maximum contig length, the number of predicted protein-coding sequences and the number of annotated genes (KEGG Orthologs, or KOs). We visualized these metrics for each material and each treatment (Fig. 1I–P) and statistically compared them between dried and frozen samples using a similar approach as for the metabarcoding metrics. In total 3 out of 8 metrics displayed a significantly negative effect of drying, 2 metrics displayed a significantly positive effect and 3 metrics did not display a significant effect (details in Table S2). The strongest positive effect of drying was observed for the maximum contig length, which was on average 33% greater for dried samples than their frozen counterparts (*P* < 0.001, n = 60) The strongest negative effect was observed in the number of contigs, with dried samples yielding on average 17% fewer contigs than their frozen counterparts (*P*=0.012, n=60). While the number of detected proteins and annotated KOs were on average lower for dried samples, these differences were not statistically significant. Thus, while upstream success metrics such as DNA yield and peak fragment size indicated substantial tradeoffs to drying samples, downstream metagenomics metrics appear less significantly impacted. This pattern is analogous to our earlier observations in metabarcoding, where the number of recovered ASVs, OTUs and genera, as well as the ASV/OTU Shannon diversities, were also not significantly different between treatments. One possible reason may be that the sequencing depth achieved for dried samples despite some DNA degradation was still sufficient to recover the bulk of detectable taxonomic and functional diversity. It is also worth pointing out the markedly different impact that drying appears to have on metagenomic and 16 S amplicon sequencing success. For example, while the number of raw amplicon reads did not differ significantly between treatments, the number of metagenomic reads was significantly lower among dried samples. These differences may be due to the greater general sensitivity of metagenomic library preparation and sequencing to DNA degradation caused during drying. Indeed, metagenomic sequencing depends on successful library conversion across the whole DNA pool [1], whereas amplicon sequencing can remain comparatively stable as long as enough short, amplifiable templates survive [56].

#### Effects on inferred gene proportions

To assess how drying affects recovered gene-centric functional profiles compared to freezing, we compared the estimated proportions of KOs between treatments similarly to how we compared OTU proportions between treatments earlier (Fig. 4). For all materials, we observed a clear and strong linear relationship between KO proportions in dried and frozen samples, with Pearson correlation coefficients ranging between 0.93 and 0.98 for feces (Fig. 4A–C) and between 0.84 and 0.87 for soils (Fig. 4D–F). Hence, dried and frozen samples indicate consistent trends in gene abundances, and this consistency is more pronounced for fecal samples. Nevertheless, in almost all cases (with the exception of feces 3) these correlations were significantly lower than expected under our null model, suggesting that desiccation significantly influenced the inferred KO proportions relative to freezing. Similar results were obtained for high-level gene groups defined according to KEGG level C (Fig. S10).

#### Effects on metagenomic $\beta$-diversity

To further quantify the effects of treatment on inferred genetic composition, we also generated MMDS plots and performed PERMANOVA tests of pairwise abundance-weighted Bray-Curtis dissimilarities in KO composition (Fig. 4G,H, Tables S6, S7 and S8). When considering only dried fecal samples or only dried soil samples, we observed a clear separation by source material, in other words, differences in KO profiles between different fecal materials or between different soils were clearly visible among dried samples (PERMANOVA $$P < 0.001 $$; Table S6). In fact, the source material explained 56% and 84% of the variance in pairwise dissimilarities among fecal or soil samples, respectively. Similar results were also obtained at the higher level of KEGG C gene groups (Figs. S10 G,H and Table S6). A significant separation by material was also observed when considering both dried and frozen samples together (Table S8 and Fig. S11). This result provides support for the use of heat-assisted sample desiccation in comparative metagenomics studies. Nevertheless, we did also observe a separation between treatments, for example when considering samples separately for each material (Fig. S12 and Table S7). Indeed, PERMANOVA revealed a significant separation between treatments for all materials except feces 3 at the level of KOs, and for all materials except feces 1 and 3 at the level of KEGG C gene groups. This separation was weaker for fecal samples than for soil samples, both in terms of the standardized effect sizes and in terms of the fraction of variance explained by the treatment. Further, when considering all fecal samples or all soil samples together, we observed a significant separation by treatment among soil samples (but not among fecal samples), both at the KO and KEGG-C level (Table S8). These observations confirm our earlier conclusion that treatment had a clear effect on inferred gene-centric metagenomic profiles, and that this effect was clearly stronger for soil samples than for fecal samples.

#### Effects on metagenome-assembled genomes

To examine the potential effects of sample drying on the recovery of prokaryotic metagenome-assembled genomes (MAGs) [55], we performed MAG binning separately for dried fecal samples, frozen fecal samples, dried soil samples, and frozen soil samples (details in the Methods). We obtained slightly more MAGs from frozen fecal compared to dried fecal samples (193 vs 182 MAGs), and slightly more MAGs from frozen soil compared to dried soil samples (55 vs 48 MAGs), although many of those MAGs were of very poor quality (Table S9). When considering only MAGs with medium or higher quality, i.e., completeness $\ge$50% and contamination $\le$10% [10], we were left with slightly more MAGs from frozen fecal compared to dried fecal samples (135 vs. 122 MAGs), but fewer MAGs from frozen soil compared to dried soil samples (6 vs 10 MAGs). For any given source type, MAG completeness and contamination levels were also similarly distributed and with nearly identical averages in frozen and dried samples (Table S9). Thus, treatment only had a small, if any, effect on the number, completeness and contamination levels of recovered MAGs.

### Caveats

We emphasize that comparative studies such as ours can only assess the effects of alternative sample preservation methods on microbiome inferences relative to each other, but not in absolute terms. Indeed, while we examined how heat-assisted desiccation followed by storage at room temperature affects microbiome inferences relative to freezing, we have not determined which treatment yielded the most accurate representation of the actual microbial communities. Answering this latter question is hard, due to numerous other sources of bias and error, for example at the DNA extraction and PCR stages [11], as well as due to variable 16 S rRNA gene copy numbers [38]. In fact, freezing and thawing can easily introduce its own taxonomic biases [17, 53].

We also note that, strictly speaking, our conclusions remain to be confirmed for other sample types, desiccation parameters, storage durations, DNA extraction protocols and so on. For example, it is possible that procedural adjustments, such as lowering the desiccation temperature at the expense of slower drying, reducing the volume of material being dried to reduce drying time, or using additional fixatives during desiccation, could decrease DNA damage [7] and increase the consistency between frozen and dried samples. Further, the duration and mode of sample storage following desiccation could also impact results, since minuscule moisture contents in the samples could enable further gradual alterations of the DNA and shifts in detectable taxa [33, 62]. In fact, the impacts of these parameters and thus also their optimal choice likely varies between sample types. While larger multifactorial experiments might in principle provide enough data to build a general model/decision tree for parameter optimization, the insights presented here serve as an encouraging starting point for researchers to optimize this protocol for their own specific samples.

Lastly, we mention that metabarcoding surveys may also target genes other than the 16 S rRNA gene considered here, most notably ITS/ITS2 (e.g., for fungi), 18 S rRNA (e.g., microeukaryotes) and CO1 (e.g., metazoa) [23, 25, 54, 59]. While we currently see no strong reason to believe that the effects of heat-assisted desiccation on sequencing success and community composition would be drastically different for these alternative loci, the precise impact remains to be investigated in future studies.

### Concluding remarks

We have explored the effects of heat-assisted sample desiccation followed by storage at room temperature on multiple aspects of common microbiome analyses, relative to conventional freezing at -80$^{\circ }$C. On the one hand, we found that dried samples generally exhibited a statistically significant deterioration in various metrics specific to DNA extraction success, such as DNA yield or peak fragment size, and in some metrics related to sequencing success, such as the number of metagenomic reads. On the other hand, these effects were less pronounced and often statistically non-significant in metrics related to downstream analysis outcomes, including the number of recovered 16 S rRNA ASVs, OTUs and genera, Shannon diversities, the number of gene KOs detected, and the number and quality of metagenome-assembled genomes. We also detected a clear impact of treatment on estimated taxon and gene proportions, especially in soil samples. However, samples from different materials (e.g., feces from different individuals) remained clearly distinguishable when considering only dried samples. This was even true when considering both dried and frozen samples together. These results suggest that heat-assisted desiccation can be an acceptable and practical sample preservation method in comparative microbiome studies, for example focusing on the impacts of fertilizer on soil microbiota or on the impacts of antibiotics on gut microbiota, when a high consistency with frozen samples is not required.

## Additional file

Supplementary file 1 (tsv 13 KB)

Supplementary file 2 (tsv 15 KB)

Supplementary file 3 (pdf 9584 KB)

## Data Availability

Raw metagenomic and amplicon sequence data are publicly available on the NCBI Sequence Read Archive under BioProject PRJNA1328827, BioSamples SAMN51361044–SAMN51361123, runs SRR35428272–SRR35428351 (metagenomes) and SRR35428585–SRR35428663 (16 S rRNA gene amplicons). Sample metadata with 16 S rRNA success metrics are available as Data File 1. Sample metadata with metagenomic success metrics are available as Data File 2.

## Abbreviations

OTU: Operational taxonomic unit
MAG: Metagenome-assembled genome
KO: KEGG Ortholog

## Code availabilitry

No new code was generated for this study. All software tools used are publicly available and mentioned in our Methods section.
