# Supplementary material for: Effects of heat-assisted sample desiccation on microbiome surveys
Source: Environ Microbiome. 2026 Apr 5;21:72. doi: 10.1186/s40793-026-00889-5 (PMC13188285; doi:10.1186/s40793-026-00889-5)
Supplement: Supplementary file 3 — (pdf 9584 KB) [file 40793_2026_889_MOESM3_ESM.pdf]

Effects of heat-assisted sample desiccation on microbiome surveys - Supplemental Information -

Claire E. Mullin[1,2] & Stilianos Louca[1,2,*]

[1]Department of Biology, University of Oregon, Eugene, USA
[2]Institute of Ecology and Evolution, University of Oregon, Eugene, USA
[*]Corresponding author

S.1 Methods details

This section provides technical details of our methods.

S.1.1 Sample collection and desiccation

Surface soil material was collected on June 6–7, 2024 from 3 different public locations in the Eugene area, including from a municipal forest and three grasslands. Feces from 3 different domestic cats were collected on during June 15–19, 2024 in the Eugene area. Soil pH was determined by mixing 20 g of soil into 50 mL of deionized water, mixing on a magnetic stirrer for 15 minutes, letting the slurry rest for another 15 minutes, then measuring using a West Tune® digital pH probe, following common practice (Kirk *et al.*, 2010; Liu *et al.*, 2013). An overview of material properties is given in Table S1, photos are provided in Fig. S2. All materials were collected in sterile tubes, and were kept at exterior temperatures in their original collection tube for about 1 day until further processing. Each material was split into 2×5 samples, of which 5 were frozen at -80°C until further processing. The remaining 5 samples were dried using heat as described below, and subsequently stored in microcentrifuge tubes inside a dark box at 25°C until further processing. We stress that the initial one-day exposure of samples to room temperature following collection does not compromise our study's conclusions, since it occurred prior to splitting, and hence its effects were shared by all samples from any given material. In fact, as all materials were collected from non-controlled environments, they had already been subjected to fluctuations in humidity, temperature and other conditions, so another day at room temperature simply extended that shared history. Replication was necessary in order to account for random effects commonly encountered in practice, including heterogeneities in the collected material, and to compare these random effects to the effects of sample treatment (drying vs. freezing). Indeed, one of our objectives was to assess how extensively sample preservation affects outcomes relative to other common sources of bias and error.

Samples were dried in 5 mL microcentrifuge tubes placed in aluminum heat blocks. Each block was heated using three ceramic Positive Temperature Coefficient (PTC) elements with a nominal terminal temperature of 70°C at 12V, attached to the sides of the block (Fig. S1A). The PTC elements of each block were powered through a digital temperature controller with a set temperature range of 60–61°C and with the controller's

temperature sensor inserted into the block's center. The entire circuit was integrated into a plastic protective case with ventilation holes drilled to the sides. Each heat block has a capacity of 28 samples and exhibits a peak power consumption of 24W (at startup) and terminal power consumption of 9.6W (once the blocks reach 60°C); thus, in total 112 samples can be dried concurrently on a regular car power supply. During the drying process, tubes were kept open but covered with PTFE filters (pore size 0.22 μm) attached to the tube mouths using Parafilm®, to allow water evaporation while preventing sample contamination (Fig. S1B). The weight of the drying samples was regularly measured using a precision scale and used to assess the progression of desiccation; desiccation was considered complete once the rate of weight loss approached zero. The final fraction of weight lost was used as an estimate of the sample's original water content (% w/w). Total desiccation times and water contents are listed in Table S1.

In March 2025, that is, approximately 9 months after sample collection and drying or freezing, DNA was extracted from all samples. DNA extractions were performed using the DNeasy™ PowerSoil Pro kit, always starting with 250 mg of material and largely following the manufacturer's protocol with the exception that samples were incubated at 50°C for 10 minutes prior to bead beating to improve overall DNA yield. Note that a brief incubation at elevated temperature is a common strategy to improve cell lysis (Moore *et al.*, 2023) that is also suggested by the manufacturer, and that the same extraction protocol was applied to all samples. Samples from the same material were always extracted on the same day, and all extractions were performed within a period of 10 days. DNA extraction yields were determined using a Qubit™ 4 fluorometer. Absorbance ratios (260 nm/280 nm an 260 nm/230 nm) were determined using an Implen™ Nanophotometer NP80. Distributions of DNA fragment sizes were determined using an Advanced Analytical™ fragment analyzer. The fragment size at the maximum density, henceforth "peak fragment size", was used as an additional extraction success metric. The above metrics are visualized in Fig. 1.

S.1.2 Statistical comparison of success metrics

To statistically evaluate the differences between dried and frozen samples with respect to each success metric (e.g., DNA yield, absorbance ratios, etc), while controlling for source material, we used a custom permutation test (Pesarin & Salmaso, 2010; Good, 2013; Louca & Mullin, 2025). Specifically, for any given metric and any given material m (feces 1–3, soil 1–3) let $X_{m1},..,X_{m5}$ denote the metric's values among the 5 dried samples and $Y_{m1},..,Y_{m5}$ the metric's values among the 5 frozen samples. We first computed the average value for the dried samples ($X_m := (X_{m1} + .. + X_{m5})/5$) and the average value for the frozen samples ($Y_m = (Y_{m1}+..+Y_{m5})/5$), and then compared these by computing their ratio $\rho_m := X_m/Y_m$. Hence, a ratio ρ_m greater or smaller than 1 means that drying tended to have a positive or negative effect, respectively, on the considered metric for the m-th material. We then computed the average of these ratios across all materials, $\rho := (\rho_1 + .. + \rho_6)/6$ in order to quantify the overall effect of drying relative to freezing (Table S2). To assess the statistical significance of this average ratio ρ, we compared it to similarly computed average ratios expected under a permutation null model. Conceptually, the null model assumes that there are no statistical differences between frozen and dried samples, while still controlling for the source material. In practice, the null model randomly permutes the samples within each material, thus breaking any associations between treatment and outcome while maintaining associations between material and outcome. We performed 10000 such permutations, and for each permutation the X_m, Y_m, ρ_m and ρ were re-computed, thus recovering the null distribution of ρ. The statistical significance of the observed ρ was computed as the fraction of null ρ that were at least as extreme as observed in either direction, i.e., with an absolute distance from 1 at least as large as observed. We emphasize that this null model specifically assesses the statistical significance of differences between treatments while fully controlling for differences between source materials. Conceptually, the above analysis is somewhat analogous to a classical two-way ANOVA with randomized block design, *response*

$\sim$ *treatment* + *material*. An advantage of this permutation approach is that we do not need to make any assumptions about the probability distribution of the data (e.g., Gaussian, constant variance etc) or about the absence or presence of interaction effects (Good, 2013). Indeed, properly designed permutation tests such as the above have been shown to be more robust than ANOVA in the case of violated assumptions (Fraker & Peacor, 2008).

S.1.3 Statistical comparison of estimated community composition

To further quantify differences in estimated OTU proportions (and similarly, ASV and genus proportions) between treatments while controlling for the material, we proceeded as follows. For any given material (e.g., soil 1), we computed for each i'th OTU its average proportion across all 5 dried samples (denoted X_i) and its average proportion across all 5 frozen samples (denoted $Y_1 i$), and then scatter-plotted these average proportions across OTUs (Figures 2A–F). Further, we computed the linear (Pearson) correlation coefficient r^2 between the log-transformed $X_1, X_2, ...$ and log-transformed $Y_1, Y_2, ...$, separately for each material. A lower r^2 indicates a lower consistency between treatments. The statistical significance (P value) was estimated relative to a permutation null model in which frozen and dried samples are statistically indistinguishable, i.e., in which treatment has no effect on community composition. In this null model, the treatment labels (frozen vs dried) associated with samples were randomly shuffled, thus breaking any association between samples and treatment. For each permutation, the average OTU proportions in each treatment and the resulting r^2 were re-computed, and the P value was set to the fraction of all 10000 permutations that yielded a lower r^2 than observed. We emphasize that this P-value is not the typical significance under a null model of zero correlation, which would correspond to the extreme situation in which the two treatments yield completely uncorrelated microbiome compositions and which would almost surely be rejected. Instead, here the P-value is the significance under a null model of no treatment effects, and thus a lower P-value indicates a more significant treatment effect. By default, only OTUs with a mean proportion $\geq 0.01\%$ in at least one compared sample were considered in this analysis, to avoid excessive noise associated with sampling stochasticity. A similar approach was used to also compare ASV proportions (Fig. S4) and genus proportions (Fig. S6) between treatments. For comparison, we also included similar analyses using all ASVs/OTUs with non-zero abundance in at least one compared sample, i.e., including rare ASVs/OTUs (Figures S5 and S3).

Table S1: **Materials overview.** Overview of materials considered. Note that from each material we collected 2×5 samples, of which 5 were frozen and 5 were dried.

**Material**	**description**	**type**	**pH**	**water content (% w/w)**	**hours dried**
feces 1	dog feces	-	-	11%	28
feces 2	feline feces	-	-	20%	28
feces 3	mule deer feces	-	-	18%	28
soil 1	surface soil, urban park	clay	5.3	6.9%	32
soil 2	surface soil, urban forest	mud/peat	5.7	22%	48
soil 3	surface soil, urban grassland	moist crumbly	5.7	11%	32

Table S2: **Statistical comparisons of success metrics.** Overview of location tests of various success metrics, comparing dried over frozen samples while controlling for the source material. Shown are the average ratio of a metric in dried samples over their frozen counterparts (averaged over all materials), the standardized effect size (SES) and the two-sided statistical significance (P-value). P-values below 0.05 are bolded. All tests are based on 60 samples, and P-values are estimated using a permutation null model described in the article. For a visual overview of metrics see Fig. 1.

**Metric**	**Average ratio dried/frozen**	**SES**	**P-value**
	DNA extraction		
DNA yield (ng)	0.438	-2.9	**0.019**
peak fragment size (bp)	0.705	-5.0	**<0.001**
260/280 ratio (nm/nm)	0.994	-1.7	0.085
260/230 ratio (nm/nm)	0.814	-2.2	**0.035**
	16S rRNA metabarcoding		
forward read quality	0.998	-0.86	0.44
reverse read quality	0.998	-2.04	**0.041**
number of read pairs	1.19	1.05	0.32
number of ASVs	0.93	-0.99	0.33
number of OTUs	0.869	-1.5	0.13
number of genera	1.07	1.5	0.10
ASV Shannon diversity	0.95	-1.4	0.17
OTU Shannon diversity	0.93	-1.8	0.071
	Metagenomics		
forward read quality	0.988	-5.2	**<0.001**
reverse read quality	1.01	+5.6	**<0.001**
number of read pairs	0.897	-2.2	**0.029**
number of contigs	0.828	-2.7	**0.012**
number of contigs $\geq$1000 bp	0.879	-1.8	0.077
max contig length (bp)	1.33	3.7	**<0.001**
number of proteins	0.831	-2.7	0.098
number of KOs	0.994	-.034	0.74

Table S3: **PERMANOVA analysis, taxonomic composition, for each material.** PERMANOVA test results, comparing abundance-weighted Bray-Curtis dissimilarities in taxonomic composition between dried and frozen samples, separately for each material, either at the level of ASVs, OTUs or genera. Shown are the pseudo F-statistic, the standardized effect size (SES), the statistical significance (P) and the fraction of variance in pairwise dissimilarities explained by the treatment (R^2). A P value below 0.05 (shown in bold) indicates systematic differences in inferred taxonomic composition between dried and frozen samples. Each test is based on 10 samples (5 dried, 5 frozen).

Material	F-statistic	SES	P	R^2
	ASV level			
feces 1	6.27	6.9	**0.0068**	0.45
feces 2	7.24	6.0	**0.0088**	0.44
feces 3	2.10	2.7	**0.023**	0.16
soil 1	7.39	7.7	**0.0017**	0.58
soil 2	10.4	8.3	**0.0034**	0.69
soil 3	5.89	7.3	**0.0055**	0.49
	OTU level			
feces 1	7.66	7.0	**0.0067**	0.49
feces 2	7.46	5.5	**0.0089**	0.40
feces 3	2.10	2.7	**0.034**	0.15
soil 1	20.5	9.6	**0.0058**	0.82
soil 2	15.3	9.0	**0.0066**	0.76
soil 3	11.1	8.5	**0.001**	0.63
	genus level			
feces 1	10.7	7.2	**0.0032**	0.54
feces 2	8.15	5.4	**0.0079**	0.39
feces 3	2.21	3.0	**0.016**	0.15
soil 1	32.3	10	**0.0078**	0.83
soil 2	17.6	9.2	**0.0069**	0.75
soil 3	15	9.0	**0.0008**	0.64

Table S4: **PERMANOVA comparison of taxonomic composition between treatments or materials, for each type.** PERMANOVA test results, comparing abundance-weighted Bray-Curtis dissimilarities in taxonomic composition between treatments (dried vs frozen) or between materials (e.g. feces 1 vs 2 vs 3, or soils 1 vs 2 vs 3), separately for each type (feces or soil) and at various taxonomic levels (ASV, OTU, genus). Shown are the pseudo F-statistic, standardized effect size (SES), the statistical significance (P) and the fraction of variance in pairwise dissimilarities explained by the grouping (R^2). Each test is based on 30 samples (either 30 fecal samples, or 30 soil samples).

**Type**	**F-statistic**	**SES**	**P**	R^2
Comparing treatments, ASV level				
feces	2.42	2.7	**0.021**	0.046
soils	5.49	10.1	**<0.001**	0.17
Comparing treatments, OTU level				
feces	2.45	2.7	**0.023**	0.042
soils	7.96	12.6	**<0.001**	0.22
Comparing treatments, genus level				
feces	2.87	3.3	**0.012**	0.047
soils	14.9	21.2	**<0.001**	0.32
Comparing source materials, ASV level				
feces	12.5	29.8	**<0.001**	0.51
soils	8.69	23.4	**<0.001**	0.47
Comparing source materials, OTU level				
feces	13.2	29.9	**<0.001**	0.49
soils	10.8	24.2	**<0.001**	0.46
Comparing source materials, genus level				
feces	12.9	27.9	**<0.001**	0.44
soils	6.59	11.6	**<0.001**	0.19

Table S5: **PERMANOVA of taxonomic composition, comparing materials, for each dried type.** PERMANOVA test results, comparing abundance-weighted Bray-Curtis dissimilarities in taxonomic composition between materials (i.e. feces 1 vs 2 vs 3) while considering only dried fecal samples or only dried soil samples, either at the level of ASVs, OTUs or genera. Shown are the pseudo F-statistic, standardized effect size (SES), the statistical significance (P) and the fraction of variance in pairwise dissimilarities explained by the grouping (R^2). Each test is based on 15 samples (either 15 fecal samples, or 15 soil samples).

**Material**	**F-statistic**	**SES**	**P**	R^2
ASV level				
dried feces	8.78	16.6	**<0.001**	0.65
dried soils	11.5	19.5	**<0.001**	0.74
OTU level				
dried feces	10.1	17.8	**<0.001**	0.67
dried soils	16.4	24.5	**<0.001**	0.77
Genus level				
dried feces	11.5	18.3	**<0.001**	0.66
dried soils	12.3	19.3	**<0.001**	0.64

Table S6: **PERMANOVA comparison of gene composition between materials, for each dried type.** PERMANOVA test results, comparing abundance-weighted Bray-Curtis dissimilarities in genetic composition between materials (i.e. feces 1 vs 2 vs 3) while considering only dried fecal samples or only dried soil samples, either at the level of KOs or KEGG-C groups. Shown are the pseudo F-statistic, standardized effect size (SES), the statistical significance (P) and the fraction of variance in pairwise dissimilarities explained by the grouping (R^2). Each test is based on 15 samples (either 15 fecal samples, or 15 soil samples).

**Treatment & type**	**F-statistic**	**SES**	**P**	R^2
	KO level			
dried feces	13.7	18.5	**<0.001**	0.56
dried soils	74.3	66.0	**<0.001**	0.84
	KEGG C level			
dried feces	13.0	16.4	**<0.001**	0.50
dried soils	140	103	**<0.001**	0.79

Table S7: **PERMANOVA comparison of gene composition between treatments, for each material.** PERMANOVA test results, comparing abundance-weighted Bray-Curtis dissimilarities in gene (KO) and KEGG-C gene group composition between dried and frozen samples, separately for each material. Shown are the pseudo F-statistic, the standardized effect size (SES), the statistical significance (P) and the fraction of variance in pairwise dissimilarities explained by the treatment (R^2). A P value below 0.05 (shown in bold) indicates systematic differences in inferred gene or gene group composition between dried and frozen samples. Each test is based on 10 samples (5 dried, 5 frozen).

**Material**	**F-statistic**	**SES**	**P**	R^2
	KO level (genes)			
feces 1	2.74	2.6	**0.027**	0.20
feces 2	11.4	8.5	**0.005**	0.57
feces 3	1.94	0.44	0.17	0.13
soil 1	61.0	13.4	**0.005**	0.95
soil 2	208	14	**0.004**	0.98
soil 3	107	14	**0.002**	0.97
	KEGG C level (gene groups)			
feces 1	2.23	2.0	0.051	0.16
feces 2	9.77	7.6	**0.004**	0.50
feces 3	2.58	0.75	0.17	0.13
soil 1	142	14	**0.004**	0.98
soil 2	464	14	**0.005**	0.97
soil 3	305	14	**0.002**	0.97

Table S8: **PERMANOVA comparison of gene composition between treatments or materials, for each type.** PERMANOVA test results, comparing abundance-weighted Bray-Curtis dissimilarities in gene (KO) or KEGG-C gene group composition between treatments (dried vs frozen) or between materials (e.g. feces 1 vs 2 vs 3, or soils 1 vs 2 vs 3), separately for each type (feces or soil). Shown are the pseudo F-statistic, standardized effect size (SES), the statistical significance (P) and the fraction of variance in pairwise dissimilarities explained by the grouping (R^2). Each test is based on 30 samples (either 30 fecal samples, or 30 soil samples).

**Type**	**F-statistic**	**SES**	**P**	R^2
	Comparing treatments, KO level			
feces	1.34	0.30	0.24	0.0097
soils	26.6	27.3	**<0.001**	0.50
	Comparing treatments, KEGG C level			
feces	1.47	0.38	0.22	0.0064
soils	37.1	33.2	**<0.001**	0.54
	Comparing source materials, KO level			
feces	16.1	19.6	**<0.001**	0.19
soils	10.3	13.4	**<0.001**	0.27
	Comparing source materials, KEGG C level			
feces	16.0	17.6	**<0.001**	0.15
soils	10.8	12.1	**<0.001**	0.23

Table S9: **Overview of MAGs.** Number of MAGs constructed for each combination of source type and treatment. Also shown are the number of MAGs of at least medium quality (completeness $\geq$50% and contamination $\leq$10%).

**Material type**	**treatment**	**NMAGs**	**Mean completeness (%)**	**Mean contamination (%)**	**NMAGs at least medium quality**
feces	dried	182	62.7	1.46	122
feces	frozen	193	63.8	1.56	135
soil	dried	48	47.6	14.0	10
soil	frozen	55	42.2	14.1	6

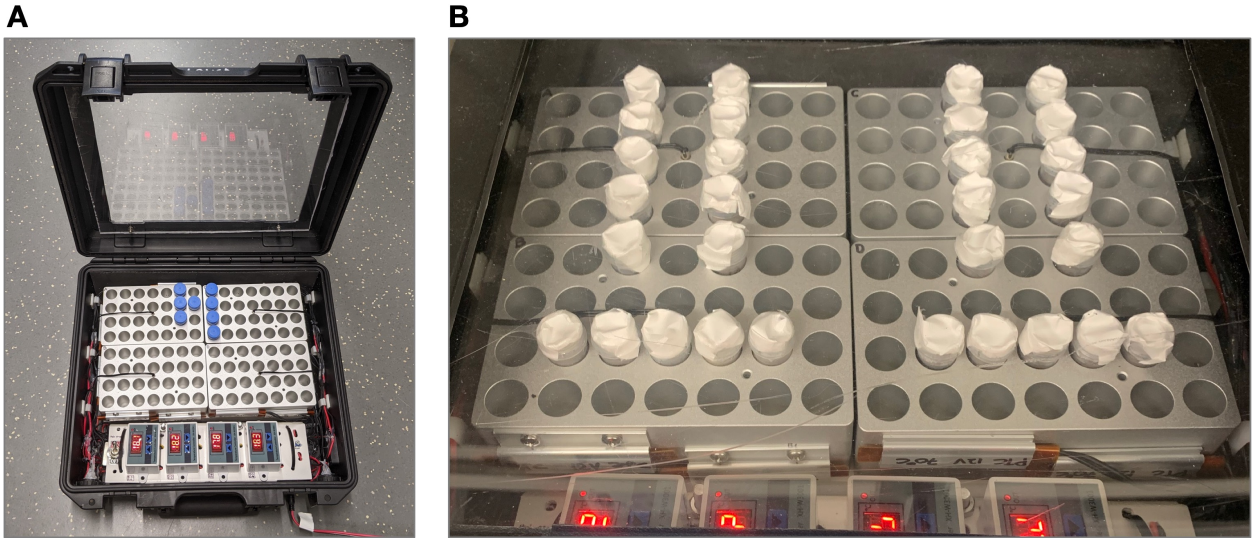

Figure S1: **Desiccator.** (A) Heat-assisted sample desiccator used in this study, essentially a block heater with thermostats integrated into a sturdy carrying case. (B) Samples being dried inside the desiccator, covered with PTFE filters (pore size 0.22 μm) to prevent contamination.

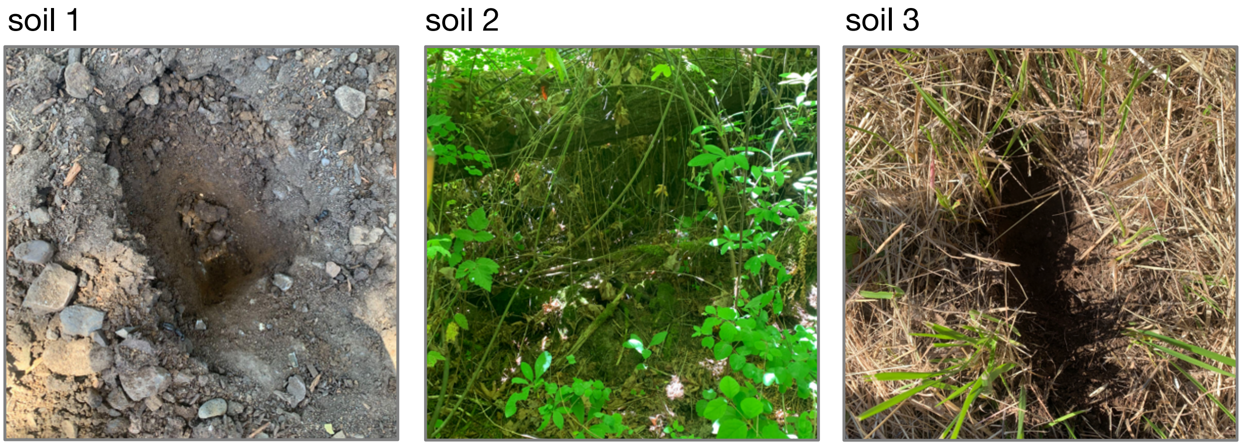

Figure S2: Photos of soil sampling locations.

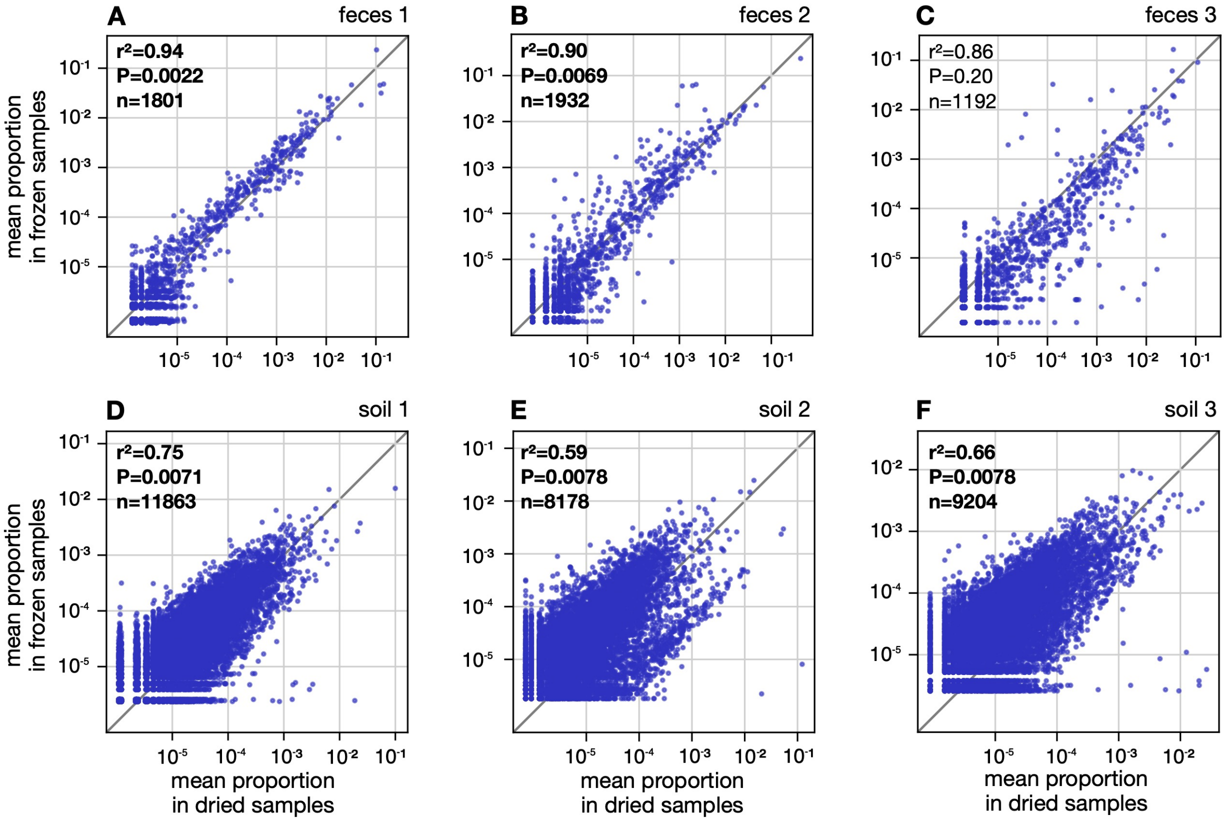

Figure S3: **OTU composition vs. treatment (including rare OTUs).** (A) Mean OTU proportions in dried fecal 1 samples (horizontal axis) compared to mean OTU proportions in frozen fecal 1 samples (vertical axis, one point per OTU), including all OTUs with non-zero abundance in at least one compared sample. Averaging of proportions was done among the 5 replicates in each treatment. The diagonal is shown for reference. Inscriptions show the Pearson correlation between the log-transformed mean OTU proportions in frozen and dried samples (r^2), the number of OTUs considered (n), and the statistical significance of r^2 compared to a permutation null model under which OTU proportions are statistically indistinguishable in the two treatments (P). Note that this null model differs from the conventional null model of zero correlation, which is extremely unrealistic and uninformative in this case. A significantly low r^2 (i.e., P<0.05) suggests that dried samples tend to yield different OTU proportions compared to frozen samples. (B–F) Similar to A, but for the remaining samples. Statistically significant r^2 values are boldened. For similar plots constrained to non-rare OTUs see Fig. 2 in the main article.

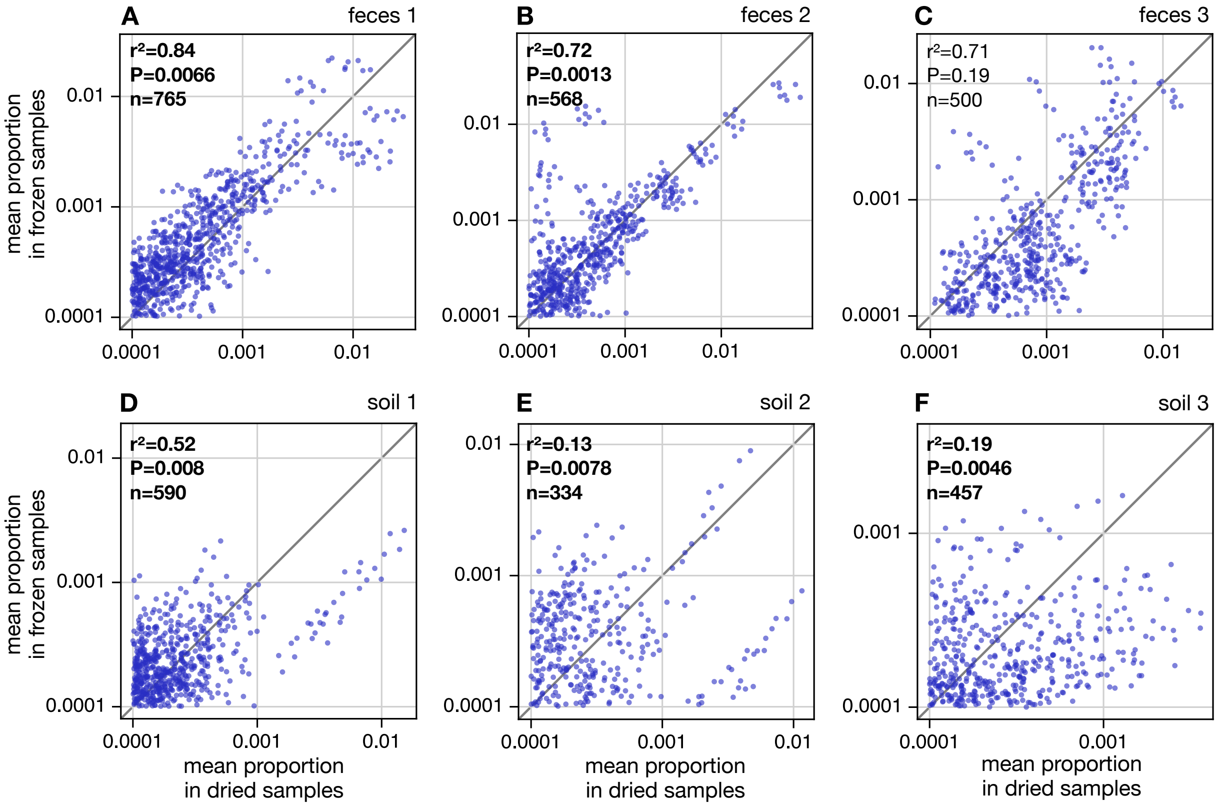

Figure S4: **ASV composition vs. treatment (abundant ASVs).** (A) Mean ASV proportions in dried fecal 1 samples (horizontal axis) compared to mean ASV proportions in frozen fecal 1 samples (vertical axis, one point per ASV), considering only ASVs with proportion $\geq$0.01% in at least one sample. Averaging of proportions was done among the 5 replicates in each treatment. The diagonal is shown for reference. Inscriptions show the Pearson correlation between log-transformed mean ASV proportions in dried and frozen samples (r^2), the number of ASVs considered (n), and the statistical significance of r^2 compared to a permutation null model under which ASV proportions are statistically indistinguishable in the two treatments (P). A significantly low r^2 (i.e., P<0.05) suggests that r^2 is lower than expected by chance, and that dried samples tend to yield different ASV proportions compared to frozen samples. (B–F) Similar to A, but for each of the other source materials. Statistically significant r^2 values are boldened. For similar plots including rare ASVs (proportion <0.01%) see Fig. S5. For similar plots using OTU or genus proportions see Figures 2 and S6, respectively.

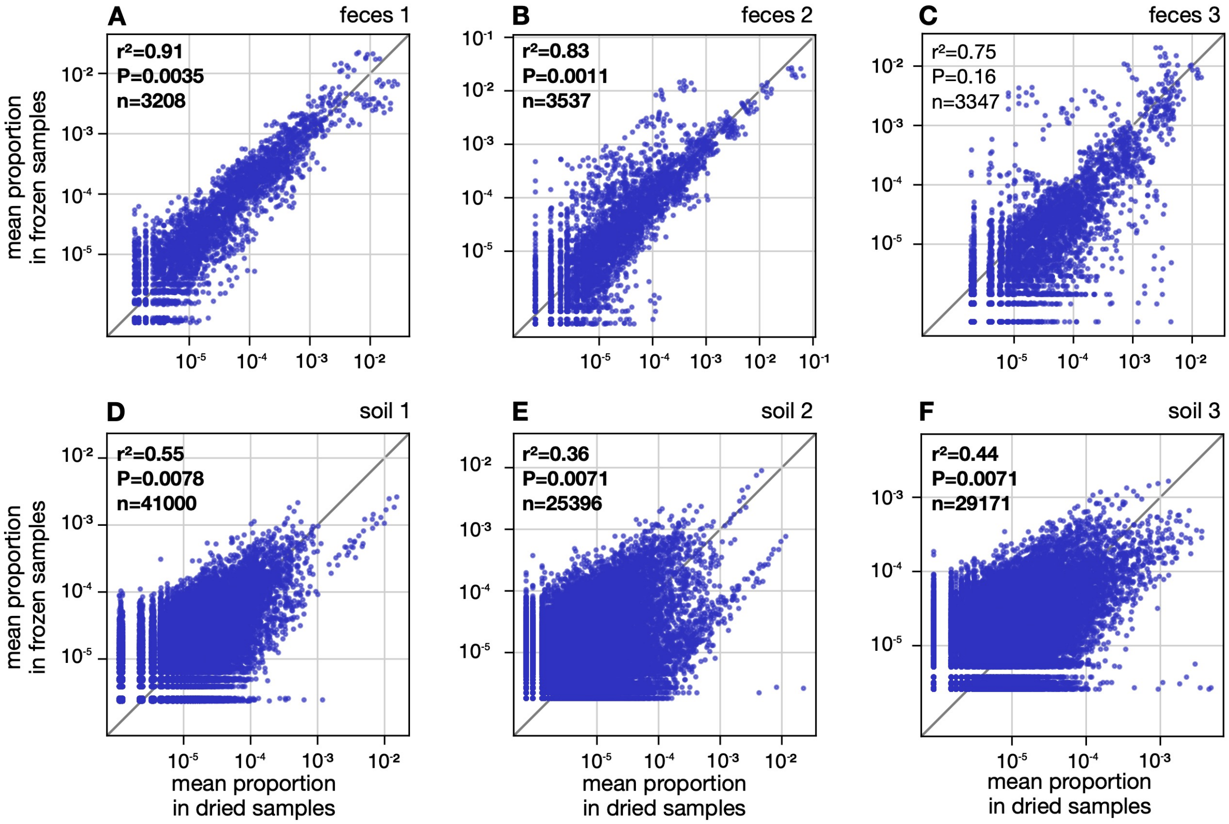

Figure S5: **ASV composition vs. treatment (including rare ASVs).** (A) Mean ASV proportions in dried fecal 1 samples (horizontal axis) compared to mean ASV proportions in frozen fecal 1 samples (vertical axis, one point per ASV), including all ASVs with non-zero abundance in at least one compared sample. Averaging of proportions was done among the 5 replicates in each treatment. The diagonal is shown for reference. Inscriptions show the Pearson correlation between log-transformed mean ASV proportions in dried and frozen samples (r^2), the number of ASVs considered (n), and the statistical significance of r^2 compared to a permutation null model under which ASV proportions are statistically indistinguishable in the two treatments (P). A significantly low r^2 (i.e., P<0.05) suggests that r^2 is lower than expected by chance, and that dried samples tend to yield different ASV proportions compared to frozen samples. (B–F) Similar to A, but for the remaining samples. Statistically significant r^2 values are boldened. (G) Metric multidimensional scaling plot of abundance-weighted Bray-Curtis dissimilarities between dried fecal samples, based on ASV proportions. Points correspond to samples, and are shaped and colored according to the source material (feces 1–3). The Kruskal stress is written in the plot. (H) Similar to G, but for dried soil samples. For a similar plot considering OTUs see Figure S3.

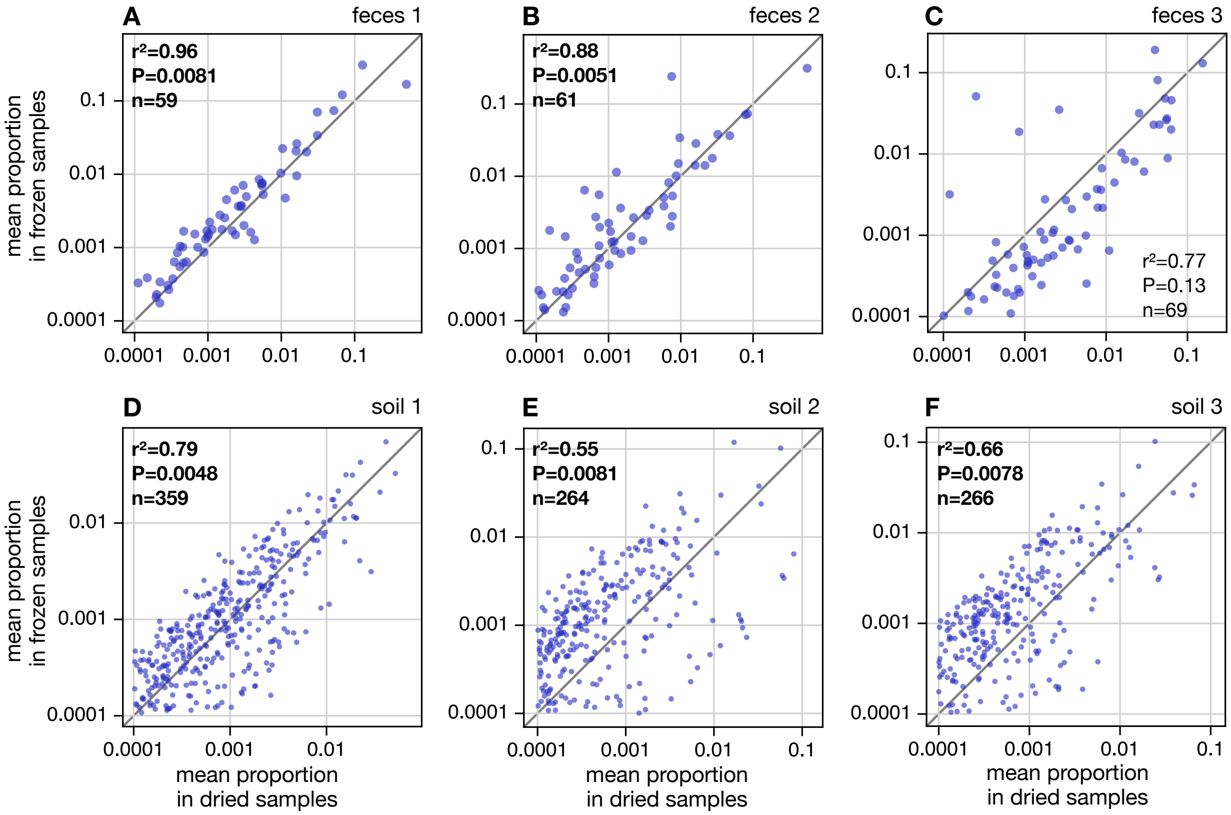

Figure S6: **Genus composition vs. treatment (abundant genera).** (A) Mean genus proportions in dried fecal 1 samples (horizontal axis) compared to mean genus proportions in frozen fecal 1 samples (vertical axis, one point per genus), considering only genera with proportion $\geq$0.01% in at least one sample. Averaging of proportions was done among the 5 replicates in each treatment. The diagonal is shown for reference. Inscriptions show the Pearson correlation between the log-transformed mean genus proportions in dried and frozen samples (r^2), the number of genera considered (n), and the statistical significance of r^2 compared to a permutation null model under which genus proportions are statistically indistinguishable in the two treatments (P). A significantly low r^2 (i.e., P<0.05) suggests that r^2 is lower than expected by chance, and that dried samples tend to yield different genus proportions compared to frozen samples. (B–F) Similar to A, but for the remaining samples. Statistically significant r^2 values are boldened. For similar plots using ASV or OTU proportions see Figures S4 and 2, respectively.

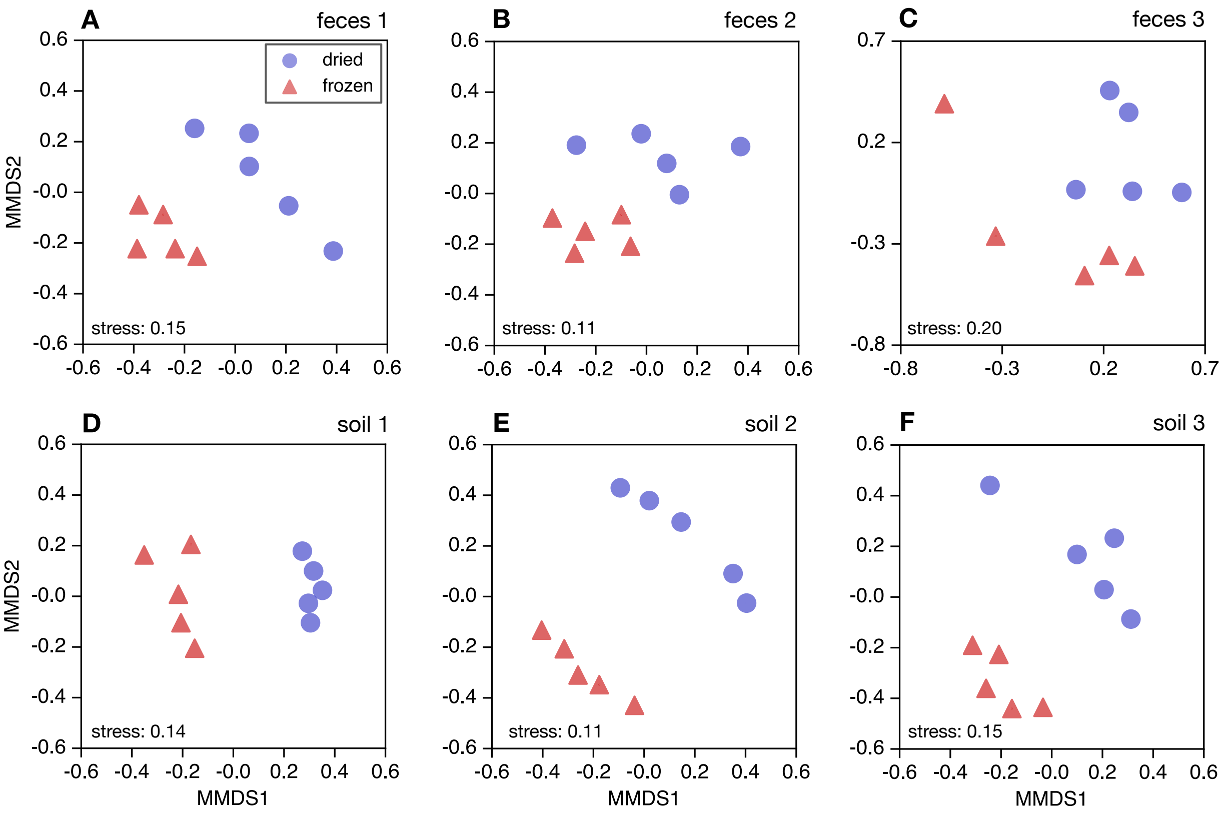

Figure S7: **Metric multidimensional scalings, separately for each material (OTU composition).** (A) Metric multidimensional scaling plot of abundance-weighted Bray-Curtis dissimilarities between "feces 1" samples, based on OTU proportions. Points correspond to samples derived from feces 1 material, and are shaped and colored according to treatment (dried vs frozen). The Kruskal stress is written in the plot. (B–F) Similar to A, but for feces 2–3 and soils 1–3. For PERMANOVA tests quantitatively examining the separation by treatment see Table S3.

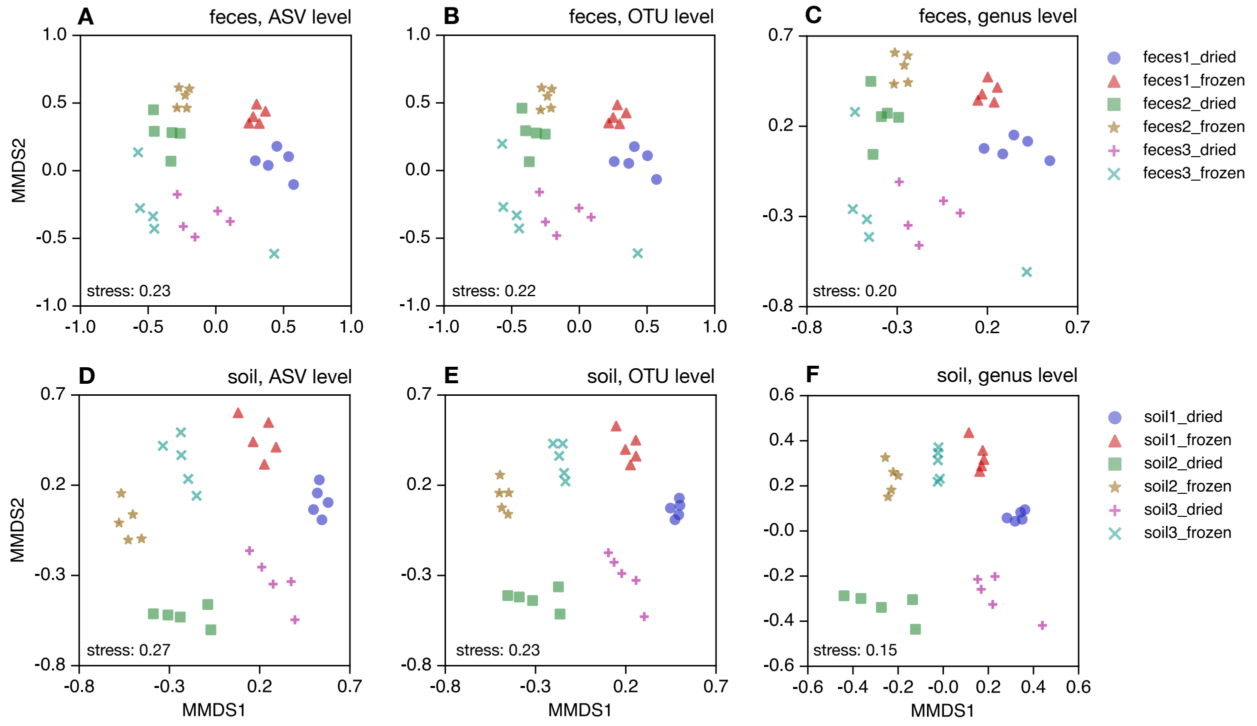

Figure S8: **Metric multidimensional scalings, separately for each type and taxonomic level.** (A–C) Metric multidimensional scaling plot of abundance-weighted Bray-Curtis dissimilarities between fecal samples, based on ASV, OTU and genus proportions, respectively. Points correspond to samples, and are shaped and colored according to the source material (feces 1–3) and treatment (dried vs frozen). The Kruskal stress is written in the plot. (D–F) Similar to A–C, but for soil samples. For PERMANOVA tests quantitatively examining the separation by treatment see Tables S3 and S4.

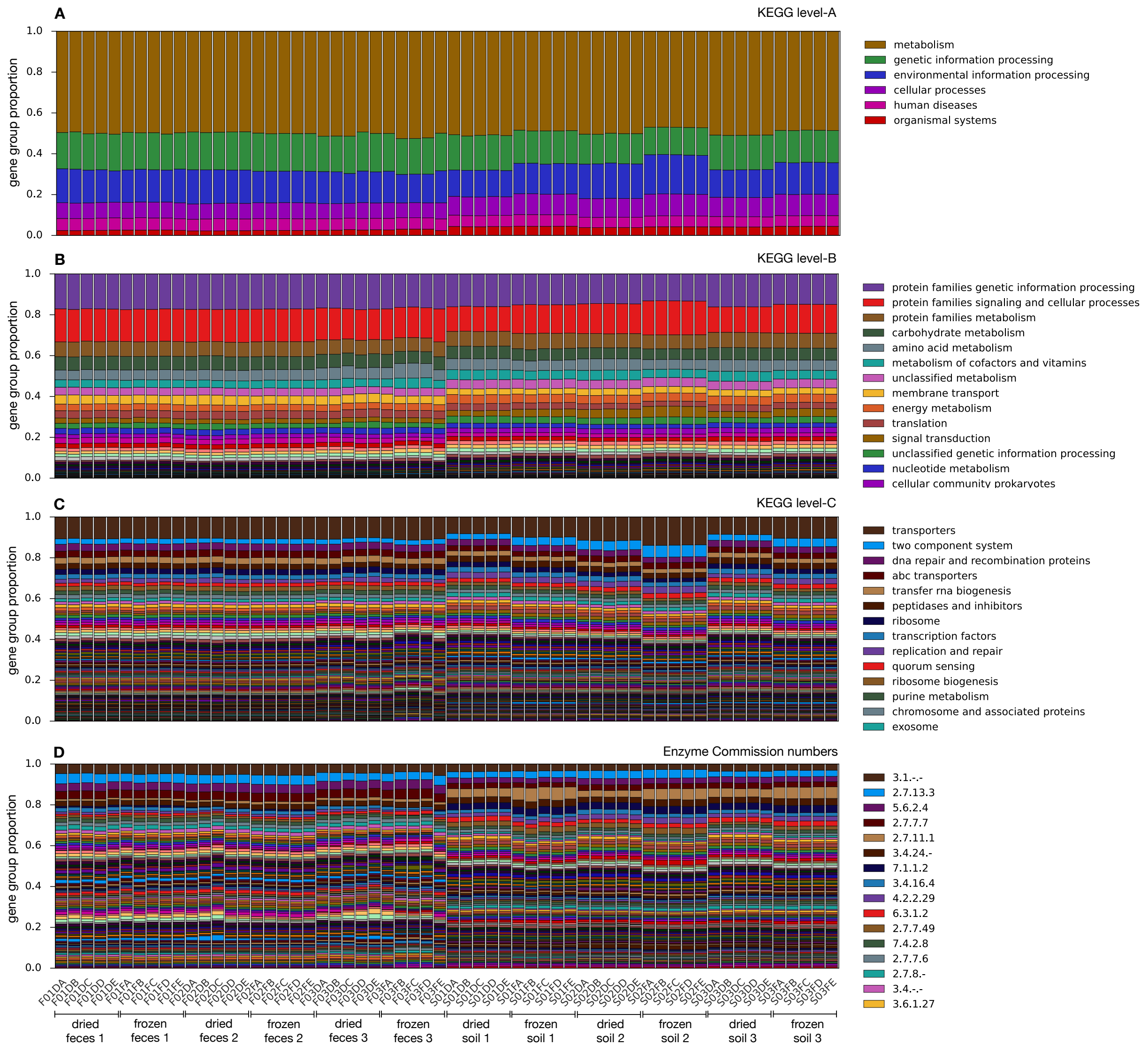

Figure S9: **KEGG gene group profiles.** (A) Estimated proportions of KEGG-level-A gene groups in each sample. Each column corresponds to a sample, and bar segment heights correspond to group proportions, which are based on numbers of reads mapped. (B, C) Similar to A, but showing KEGG level-B and KEGG level-C groups, respectively. (D) Similar to A, but showing gene groups representing distinct enzyme commission numbers. In B–D, only the top 100 groups are shown and only a subset of these is included in the legend due to space limitations.

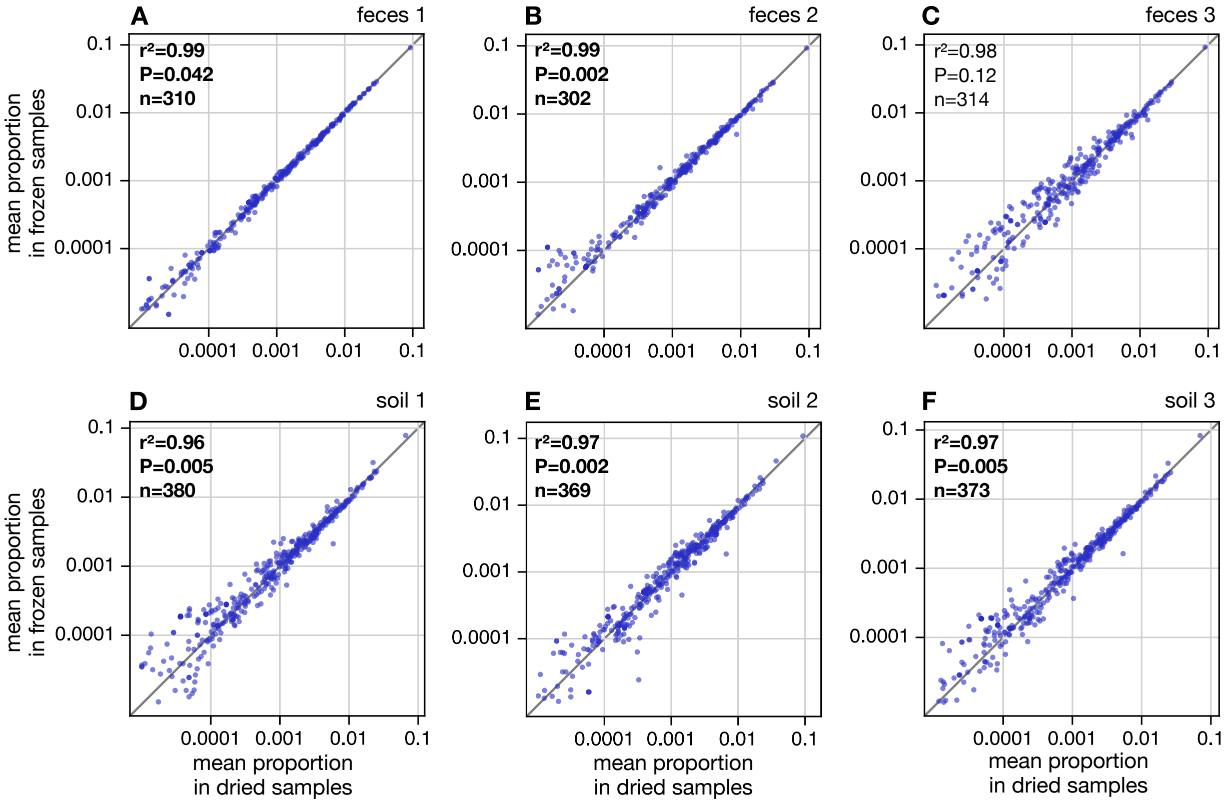

Figure S10: **KEGG C composition vs. treatment.** (A) Mean KEGG C gene group proportions in dried fecal 1 samples (horizontal axis) compared to mean gene group proportions in frozen fecal 1 samples (vertical axis, one point per gene group), considering only gene groups with proportion $\geq$0.001% in at least one compared sample. Averaging of proportions was done among the 5 replicates in each treatment. The diagonal is shown for reference. Inscriptions show the Pearson correlation between log-transformed mean gene group proportions in dried and frozen samples (r^2), the number of gene groups considered (n), and the statistical significance of r^2 compared to a permutation null model under which gene group proportions are statistically indistinguishable in the two treatments (P). A significantly low r^2 (i.e., P<0.05) suggests dried samples tend to yield different gene group proportions compared to frozen samples. (B–F) Similar to A, but for the remaining samples. Statistically significant r^2 values are boldened. For similar plots using gene (KO) proportions see Fig. 4.

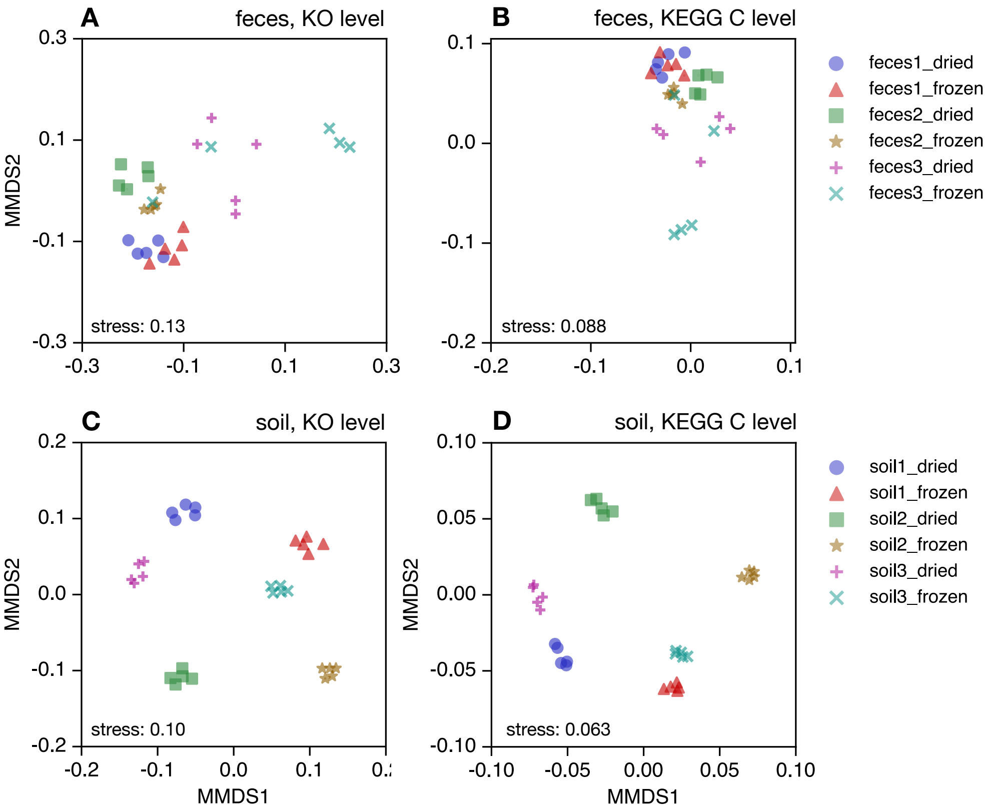

Figure S11: **Metric multidimensional scalings, separately for each type and KEGG level.** (A,B) Metric multidimensional scaling plot of abundance-weighted Bray-Curtis dissimilarities between fecal samples, based on KO and KEGG-C group proportions, respectively. Points correspond to samples, and are shaped and colored according to the source material (feces 1–3) and treatment (dried vs frozen). The Kruskal stress is written in the plot. (C,D) Similar to A,B, but for soil samples. For PERMANOVA tests quantitatively examining the separation by treatment see Tables S7 and S8.

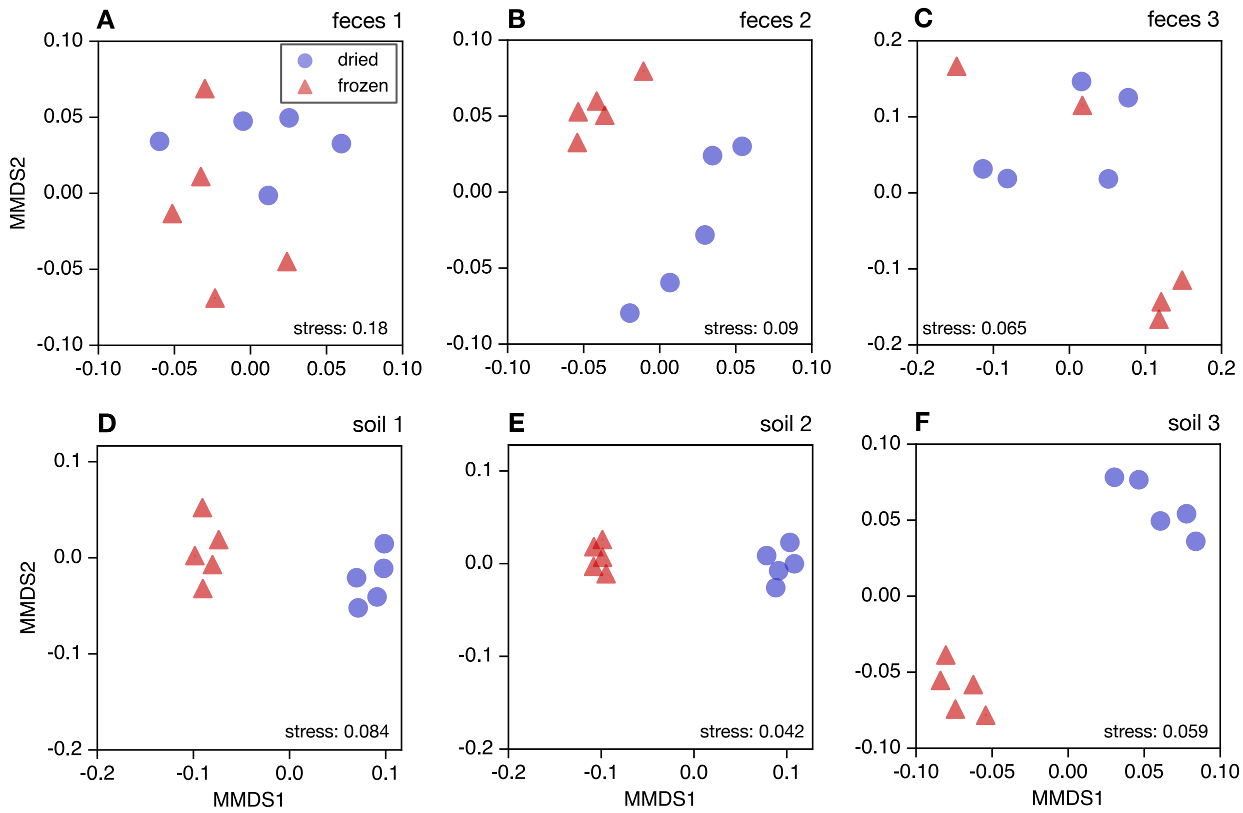

Figure S12: **Metric multidimensional scalings, separately for each material (KO composition).** (A) Metric multidimensional scaling plot of abundance-weighted Bray-Curtis dissimilarities between "feces 1" samples, based on KO proportions. Points correspond to samples derived from feces 1 material, and are shaped and colored according to treatment (dried vs frozen). The Kruskal stress is written in the plot. (B–F) Similar to A, but for feces 2–3 and soils 1–3. For PERMANOVA tests quantitatively examining the separation by treatment see Table S7.

References

Fraker, M.E. & Peacor, S.D. (2008) Statistical tests for biological interactions: A comparison of permutation tests and analysis of variance. *Acta Oecologica*, 33, 66–72.

Good, P. (2013) Permutation tests: a practical guide to resampling methods for testing hypotheses. New York, NY: Springer.

Kirk, G.J.D., Bellamy, P.H., & Lark, R.M. (2010) Changes in soil pH across England and Wales in response to decreased acid deposition. *Global Change Biology*, 16, 3111–3119.

Liu, Z.P., Shao, M.A., & Wang, Y.Q. (2013) Large-scale spatial interpolation of soil pH across the Loess Plateau, China. *Environmental Earth Sciences*, 69, 2731–2741.

Louca, S. & Mullin, C.E. (2025) Effects of vacuum-heat-assisted sample desiccation on microbiome surveys. *Molecular Ecology Resources*, 25, e70020.

Moore, M.A., Scheible, M.K.R., Robertson, J.B., & Meiklejohn, K.A. (2023) Assessing the lysis of diverse pollen from bulk environmental samples for dna metabarcoding. *Metabarcoding and Metagenomics*, 6, e89753.

Pesarin, F. & Salmaso, L. (2010) The permutation testing approach: a review. *Statistica*, 70, 481–509.
